# Human Immunodeficiency Virus (HIV)–Infected CCR6^+^ Rectal CD4^+^ T Cells and HIV Persistence On Antiretroviral Therapy

**DOI:** 10.1093/infdis/jiz509

**Published:** 2019-12-04

**Authors:** Jenny L Anderson, Gabriela Khoury, Rémi Fromentin, Ajantha Solomon, Nicolas Chomont, Elizabeth Sinclair, Jeffrey M Milush, Wendy Hartogensis, Peter Bacchetti, Michael Roche, Carolin Tumpach, Matthew Gartner, Matthew C Pitman, Christine Lorrie Epling, Rebecca Hoh, Frederick M Hecht, Ma Somsouk, Paul U Cameron, Steven G Deeks, Sharon R Lewin

**Affiliations:** 1 The Peter Doherty Institute for Infection and Immunity, The University of Melbourne and Royal Melbourne Hospital, Melbourne, Victoria, Australia; 2 Centre de Recherche du CHUM and Department of Microbiology, Infectiology and Immunology, Université de Montréal, Montreal, Quebec, Canada; 3 Department of Medicine, University of California San Francisco, San Francisco, California, USA; 4 Department of Epidemiology and Biostatistics, University of California, San Francisco, California, USA; 5 School of Health and Biomedical Sciences, RMIT University, Melbourne, Victoria, Australia; 6 Department of Infectious Diseases, Alfred Hospital and Monash University, Melbourne, Victoria, Australia

**Keywords:** HIV reservoir, latency, persistence, chemokine receptor, CCR6, CXCR3, chemokines, rectal tissue, lymph node

## Abstract

**Background:**

Identifying where human immunodeficiency virus (HIV) persists in people living with HIV and receiving antiretroviral therapy is critical to develop cure strategies. We assessed the relationship of HIV persistence to expression of chemokine receptors and their chemokines in blood (n = 48) and in rectal (n = 20) and lymph node (LN; n = 8) tissue collected from people living with HIV who were receiving suppressive antiretroviral therapy.

**Methods:**

Cell-associated integrated HIV DNA, unspliced HIV RNA, and chemokine messenger RNA were quantified by quantitative polymerase chain reaction. Chemokine receptor expression on CD4^+^ T cells was determined using flow cytometry.

**Results:**

Integrated HIV DNA levels in CD4^+^ T cells, CCR6^+^CXCR3^+^ memory CD4^+^ T-cell frequency, and CCL20 expression (ligand for CCR6) were highest in rectal tissue, where HIV-infected CCR6^+^ T cells accounted for nearly all infected cells (median, 89.7%). Conversely in LN tissue, CCR6^+^ T cells were infrequent, and there was a statistically significant association of cell-associated HIV DNA and RNA with CCL19, CCL21, and CXCL13 chemokines.

**Conclusions:**

HIV-infected CCR6^+^ CD4^+^ T cells accounted for the majority of infected cells in rectal tissue. The different relationships between HIV persistence and T-cell subsets and chemokines in rectal and LN tissue suggest that different tissue-specific strategies may be required to eliminate HIV persistence and that assessment of biomarkers for HIV persistence may not be generalizable between blood and other tissues.

## Teaser

The relationship between human immunodeficiency virus (HIV) persistence and proportions of CD4^+^ T cells expressing various chemokine receptors or their chemokines differs among anatomic sites. Rectal CCR6^+^ CD4^+^ T cells have a major contribution to the HIV reservoir in recipients of antiretroviral therapy.

Human immunodeficiency virus (HIV) persists among people living with HIV (PLWH) and receiving suppressive antiretroviral therapy (ART) in resting and proliferating memory CD4^+^ T cells in blood and tissue [1–4]. In humans, <3% of T cells reside in peripheral blood, whereas most T cells inhabit secondary lymphoid tissues, including lymph nodes (LN) and gut-associated lymphoid tissue (GALT) [5, 6]. In PLWH on suppressive ART, HIV DNA is enriched in CD4^+^ T cells from GALT and LN tissue compared to blood [7–10]. Infected T cells can also have a spectrum of transcriptional activity [11] from truly latent (no transcription) to higher HIV RNA expression, which is more common in lymphoid tissue [9, 10, 12]. Dissecting the factors associated with HIV persistence and transcription in tissue in PLWH receiving ART is important for developing new cure strategies.

Chemokines and their chemokine receptors (CKRs) mediate trafficking of leukocytes to specific tissues [13]. CCL19 and CCL21 chemokines, produced by stromal cells in lymphoid organs and lymphatic endothelial cells, bind CCR7 on naive and central memory T cells, promoting migration to lymphoid tissue [14]. CCL20 chemokine, produced by various epithelial cell types, binds CCR6 on T-helper (Th) 17 cells and other populations, inducing homing to the gastrointestinal tract [15]. CXCL9, CXCL10, and CXCL11 chemokines, secreted by monocytes, endothelial cells, and fibroblasts in response to interferon γ, bind CXCR3-expressing Th1 cells, promoting migration to inflammatory sites [16]. CCL19, CCL20, and CXCL9/CXCL10 levels are elevated in plasma during untreated HIV infection [17, 18].

HIV replicates in activated CD4^+^ T cells that express CCR6 and high levels of the HIV coreceptor CCR5 [19–22]. In untreated HIV infection, CCR6^+^ CD4^+^ T cells are targets of HIV and simian immunodeficiency virus (SIV) replication in the GALT [23–26] and female reproductive tract [27]. CKRs are also important to viral persistence in PLWH receiving ART. In blood, HIV persists in CD4^+^ T cells that express CKRs CXCR3, CCR6, and CCR7 [28, 29], and inducible replication-competent virus is enriched in CXCR3^+^ CD4^+^ T cells receiving ART [30]. In LN tissue, HIV persists in PD1^+^CXCR5^+^ T follicular helper cells in B-cell follicles during ART [9], and in an SIV infection model, SIV persists in CD4^+^ regulatory T (Treg) cells that express cytotoxic T lymphocyte antigen 4 and high CCR6 [31]. In GALT, CCR6^+^ CD4^+^ T cells have higher HIV Gag DNA than CCR6− T cells during ART [29]. Finally, in vitro, CCL19, CCL20 or CXCL10 chemokines also enhance latent HIV infection [32, 33], suggesting that high levels of these chemokines in tissue may favor the establishment of latent infection at these sites.

To examine whether expression of CCR6, CXCR3, CCR7, and CCR5 CKRs and their chemokines were associated with HIV persistence in different anatomic sites, we studied blood, rectal, and LN tissue from PLWH taking ART in a cross-sectional study. We found that HIV was enriched in CD4^+^ T cells from rectal tissue versus blood. In rectal tissue, a high proportion of CD4^+^ T cells expressed CCR6 and CXCR3, CCL20 chemokine expression was increased, and HIV-infected CCR6^+^ T cells accounted for nearly all of the total infected cells. Conversely in LN tissue, CCR6^+^ T cells were infrequent, and there was a significant association between HIV persistence and expression of CCL19, CCL21, and CXCL13 chemokines. The different relationships between HIV persistence and T-cell subsets in blood, rectal tissue, and LN tissue suggests that different tissue-specific strategies may be required to eliminate HIV persistence during ART. These findings also suggest that blood-based biomarkers may not accurately characterize the total HIV reservoir in tissue.

## METHODS

### Study Participants

PLWH taking suppressive ART (HIV RNA, <40 copies/mL) for ≥3 years with CD4^+^ T-cell counts >350/μL were recruited at the University of California, San Francisco, and are described elsewhere [10, 28, 34]. Participants provided informed consent before participation. The study was approved by the University of California, San Francisco (institutional review board no. 10-01330), and by Monash University and the University of Melbourne (human research ethics committees nos. 2012000032 and 1443162, respectively) in Australia.

### Sample Processing and Analyses

Participant blood, LN, and rectal tissue samples were assessed for HIV DNA and RNA in sorted total CD4^+^ or CCR6^+^/CXCR3^+^ T-cell subsets, CKR immunophenotyping, or chemokine or transcription factor RNA (Supplementary Material). In statistical analyses, *P* values < .05 were considered statistically significant, and nominal *P* values were reported without adjustment for multiple comparisons, as outlined elsewhere [34] (Supplementary Material).

## RESULTS

### Enrichment of HIV in CD4^+^ T Cells From Rectal Tissue Compared With Blood

A primarily male cohort treated with suppressive ART was recruited, with a median (interquartile range [IQR]) age of 57 (50–62) years (Table 1). Median (IQR) nadir and current CD4^+^ T-cell counts were 216/μL (133–387/μL) and 684/μL (530–862/μL) cells respectively.

**Table 1.** Demographic Characteristics of Study Participants

Parameter	Full Cohort With Blood Samples (n = 48)	Subgroup With LN Samples (n = 8)	Subgroup With Rectal Samples (n = 20)
Age, median (IQR), y	56.5 (50–62)	57.5 (50–62)	58.5 (51–63)
Sex, no. (%)			
Male	46 (96)	8	20
Female	1 (2)	0	0
Transgender^a^	1 (2)	0	0
Race, no. (%)			
White	32 (67)	6 (75)	13 (65)
African	6 (13)	1 (13)	1 (5)
Hispanic	4 (8)	0 (0)	2 (10)
Asian	1 (2)	0 (0)	0 (0)
Pacific Islander	1 (2)	0 (0)	1 (5)
Mixed	4 (8)	1 (13)	3 (15)
Duration of ART, median (IQR), y	8.5 (5.0–12.4)	10.9 (4.7–13.1)	10.5 (5.3–12.5)
Viral load, copies/mL	<40	<40	<40
CD4^+^ T-cell count, median (IQR)			
Nadir, cells/μL	216 (133–387)	134 (80–383)	143 (89–387)
Current, cells/μL	684 (530–862)	549 (429–719)	669 (496–833)
Current, %	32 (25–41)	25 (21–31)	30 (23–36)
Current CD8^+^ T-cell count, median (IQR), cells/μL	914 (639–1091)	1069 (893–1399)	986 (745–1138)
Integrated HIV DNA, median (IQR), copies/10^6^ T cells	338.8 (153.4–700.9) (n = 48)	449.7 (217.1–1850) (n = 7)	1263 (413.5–1942) (n = 19)
HIV CA-US RNA, median (IQR), copies/10^6^ T cells	22.5 (11.8–38.6) (n = 44)	75.3 (13.9–155.8) (n = 7)	69.4 (18.3–301.3) (n = 16)
Ratio of HIV CA-US RNA to integrated DNA, median (IQR)	0.065 (0.037–0.122) (n = 43)	0.086 (0.041–0.328) (n = 7)	0.041 (0.029–0.201) (n = 13)

Abbreviations: ART, antiretroviral therapy; HIV, human immunodeficiency virus; IQR, interquartile range.

^a^Transgender woman, with use of exogenous estrogens unknown.

Quantification of HIV persistence revealed that total CD4^+^ T cells from rectal tissue had significantly higher integrated HIV DNA levels than cells from blood or LN tissue (fold difference, 3.91 [*P* < .001] and 2.42 [*P* = .01], respectively) (Supplementary Table 1] as previously published for this cohort [10]. HIV CA-US RNA levels were also higher in CD4^+^ T cells from rectal and LN tissue than in those from blood (fold difference, 4.57 and 3.66, respectively; both *P* < .001) (Supplementary Table 1 [10]). However, there was no statistically significant difference between the 3 anatomic sites in the ratio of CA-US RNA to integrated DNA (Supplementary Table 1).

**Figure 1.**
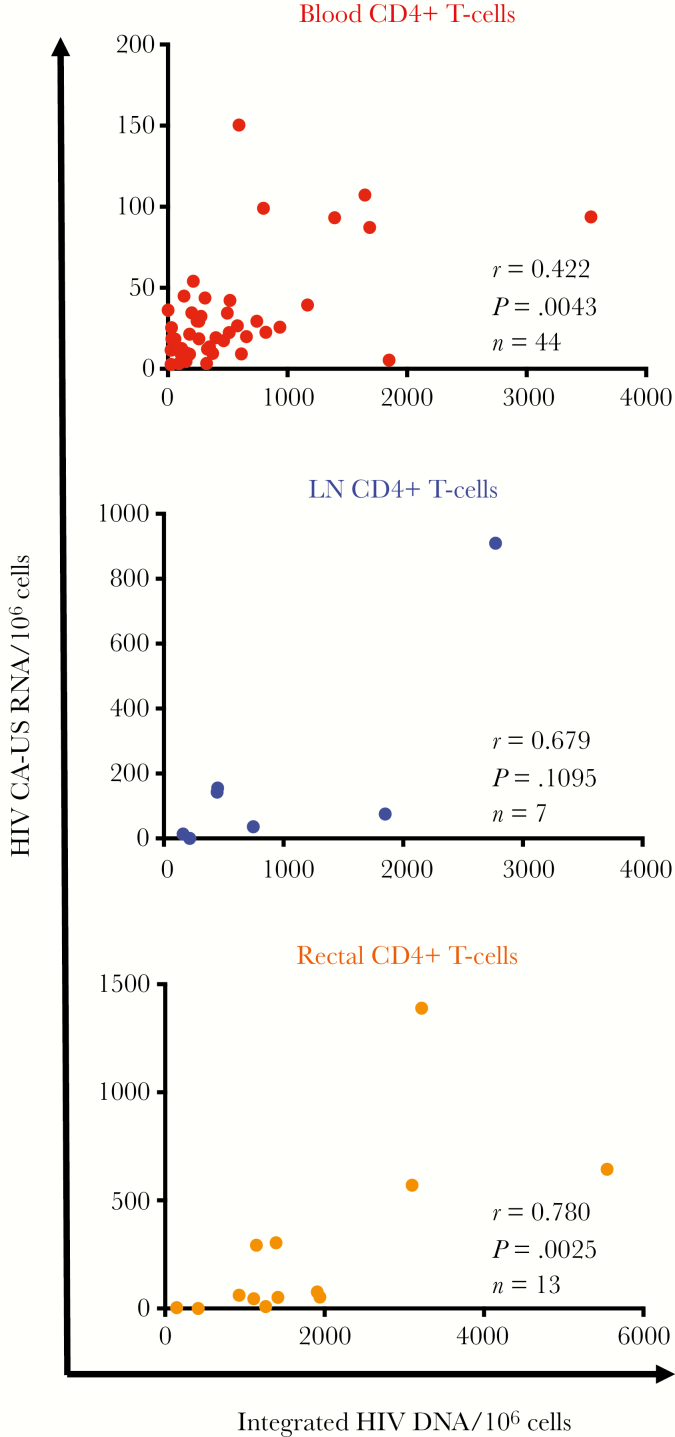
Positive correlation between human immunodeficiency virus (HIV) integrated DNA and CA-US RNA in total CD4^+^ T cells from blood and rectal tissue. Figure displays integrated HIV DNA and cell-associated unspliced RNA (CA-US RNA) levels in total CD4^+^ T cells from peripheral blood (n = 44; *top*), lymph node (LN) tissue (n = 7; *middle*) and rectal tissue (n = 13; *bottom*) from people living with HIV and receiving antiretroviral therapy.

Integrated HIV DNA and CA-US RNA levels were positively correlated in blood and rectal CD4^+^ T cells (*P* = .004 and *P* = .003, respectively), but the positive correlation did not reach statistical significance in cells from LN tissue (Figure 1). There were no statistically significant correlations between markers of HIV persistence and different anatomic sites. These findings may be a consequence of the fewer LN samples obtained (Supplementary Table 2).

Single-genome amplification and sequencing of the HIV *env* gene using CD4^+^ T cells from peripheral blood, LN tissue, or rectal tissue for 5 participants revealed occasional identical HIV *env* sequences in blood and LN or rectal tissue (Supplementary Figure 1) and we also found genetically distinct sequences between compartments. There was no evidence of compartmentalization (Supplementary Table 3).

### Enrichment of Memory CD4^+^ T Cells Coexpressing CCR6, CXCR3, and CCR5 in Rectal Tissue

The distribution of total memory CD4^+^ T cells that express single CKRs or combinations of CKRs were examined in the 3 anatomic sites. CCR7 was excluded from analysis owing to lost staining intensity over the duration of processing. CD45RA^+^CD27^+^ naive T cells were also excluded from analysis because rectal tissue has minimal naive T cells but blood and LN tissues are enriched in them.

**Figure 2.**
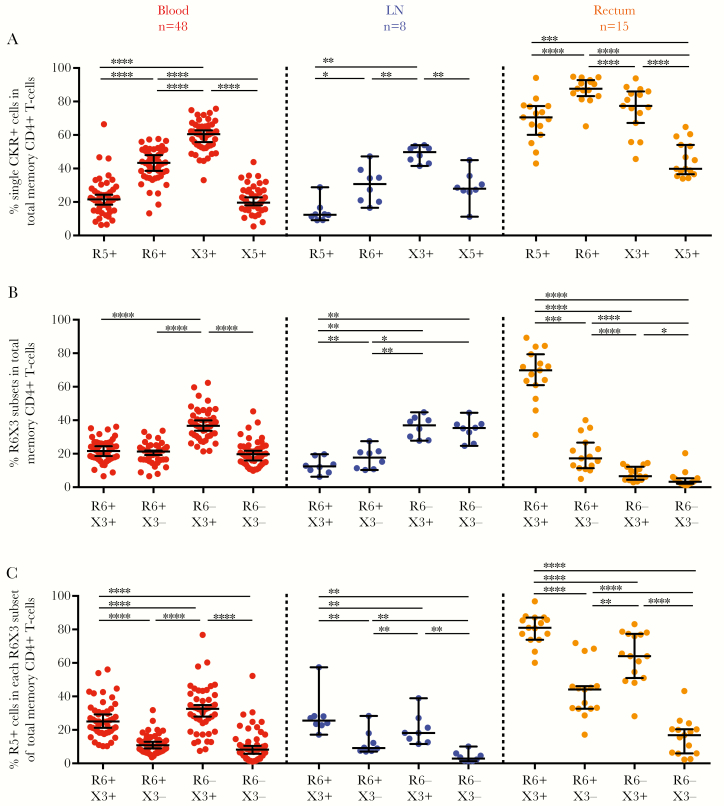
Rectal tissue harbors a high proportion of total memory CD4^+^ T cells that coexpress CCR6, CXCR3, and CCR5. Single-cell suspensions were isolated from peripheral blood (left panel), lymph node (LN) (middle panel) tissue, or rectal tissue (right panel) from people living with human immunodeficiency virus (HIV) and receiving antiretroviral therapy. Cells were then stained and analyzed with flow cytometry for expression of CD14, CD19, CD3, CD4, CD45RA, CD27, CCR5, CCR6, CXCR3, and CXCR5. *A, B,* Proportion of total memory CD4^+^ T cells that express single CKRs (*A*) or CCR6 and/or CXCR3 (*B*). *C,* Percentage of cells expressing the CCR5 HIV entry coreceptor in each CCR6 and/or CXCR3 subset. Data are displayed as median with interquartile range for each anatomic site, and dots represent individual donors. Differences between cell types were determined using the Wilcoxon matched-pairs signed rank test, with significance defined as *P* < .05. *P < .05; **P < .01, ***P < .005; **** *P* < .001. Abbreviations: R5, CCR5; R6, CCR6; X3, CXCR3; X5, CXCR5.

In single-CKR analyses (Figure 2), most rectal memory CD4^+^ T cells expressed CCR6, CXCR3, or CCR5, and a smaller proportion expressed CXCR5 (median, 87.6%, 77.4%, and 70.5% vs 39.8%, respectively). Because the expressed CKRs are not mutually exclusive, the proportions add up to >100%. A different profile was observed for blood and LN tissue, where the frequency of cells expressing a single CKR was lower than in rectal tissue (Figure 2A).

When coexpression of CKRs was analyzed, rectal tissue contained a high proportion of CCR6^+^CXCR3^+^ memory cells (median, 69.8%) (Figure 2B) that frequently coexpressed CCR5 (81.0%) (Figure 2C). Conversely, in blood and LN tissue, the frequencies of CCR6^+^CXCR3^+^ T cells were lower (median, 21.6% and 12.4%, respectively) (Figure 2B). CXCR3^+^ T-cell subsets at all 3 sites exhibited greater CCR5 coexpression than CXCR3^−^ T-cell subsets (Figure 2C).

### Transcriptional Features of T Cells Expressing CCR6 and CXCR3

CD4^+^ T cells can also be classified based on function. This includes Th17 (CCR6^+^), Treg (either CCR6^+^ or CXCR3^+^), and Th1 (CXCR3^+^) subsets that are distinguished by expression of the transcription factors RORγT, FoxP3, and T-bet, respectively [35–38]. To confirm the functional subsets in the T-cell populations quantified according to CKR expression, we also quantified the messenger RNA (mRNA) transcript levels of RORγT, FoxP3, and T-bet. Transcript levels in sorted T cells from participants were expressed relative to levels in a calibrator sample, here being RNA from peripheral blood mononuclear cells collected from a single healthy donor (Supplementary Material).

**Figure 3.**
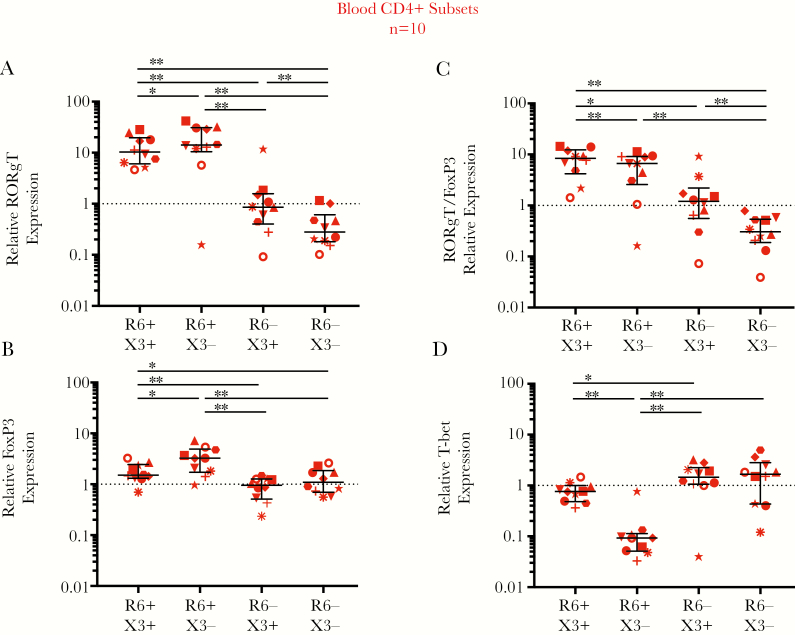
Relative expression of RORγT, FoxP3, and T-bet transcription factors in blood CCR6/CXCR3 memory T-cell subsets. The relative expression of T-cell transcription factor messenger RNAs (mRNAs) was assessed in CCR6/CXCR3 CD8^−^ memory T-cell subsets sorted from the blood of 10 people living with human immunodeficiency virus and receiving antiretroviral therapy (ART), using quantitative reverse-transcription polymerase chain reaction. Transcription factors include RORγT, which marks T-helper (Th) 17 cells (*A*); FoxP3, which marks regulatory T (Treg) cells (*B*); the ratio of RORγT to FoxP3, which marks the abundance of Th17 to Treg cells (*C*); and T-bet, which marks Th1 cells (*D*). The relative expression of transcription factor mRNA in the blood T-cell subsets is shown relative to levels in a calibrator sample, here being RNA from peripheral blood mononuclear cells collected from a single healthy donor (*dotted line at 1*). Data are displayed as median and interquartile range, and each symbol represent an individual donor. Differences between cell types were assessed using the Wilcoxon matched-pairs signed rank test, with significance defined as *P* < .05. *P < .05; **P < .01. Abbreviations: R6, CCR6; X3, CXCR3.

In blood, CCR6^+^CXCR3^+^ and CCR6^+^CXCR3^−^ memory T cells had a statistically significant higher expression of RORγT (Th17) and FoxP3 (Treg) and ratio of RORγT to FoxP3 than both CCR6^−^ populations (Figure 3A–3C), suggesting that blood CCR6^+^ T cells are enriched for Th17 cells. CCR6^+^CXCR3^+^ cells had a higher T-bet (Th1) expression than CCR6^+^CXCR3^−^ cells, consistent with both Th1Th17 and Th17 functions (Figure 3D). In rectal tissue, there were insufficient cells to sort CCR6 and/or CXCR3 populations, so only total CD4^+^ T cells were assessed. Rectal CD4^+^ T cells had 1.95-fold higher median expression of RORγT relative to FoxP3 (*P* < .001; Supplementary Figure 2), consistent with Th17 cells, similar to findings in blood CCR6^+^ T cells.

### Different Relationships Between HIV Persistence and Frequencies of CKR Memory CD4^+^ T Cells in Rectal Versus LN Tissue

We further examined whether HIV persistence was associated with frequencies of memory T-cell subsets expressing CKRs. Negative binomial regression models were used with and without adjustment for potential confounding effects of current and/or nadir CD4^+^ T-cell count, as published elsewhere [28, 34, 39] (Supplementary Material).

In rectal tissue, integrated HIV DNA and CA-US RNA levels and the RNA-to-DNA ratio had a statistically significant positive relationship with the frequency of CCR6^+^CXCR3^−^ T cells in unadjusted (fold difference, 1.04 [95% confidence interval (CI), 1.01–1.09] [*P* = .02], 1.08 [1.01–1.16] [*P* = .03], and 1.06 [1.01–1.11] [*P* = .02], respectively) and adjusted models (Table 2). In contrast, integrated HIV DNA and CA-US RNA had a statistically significant inverse relationship with CCR6^+^CXCR3^+^ T-cell frequency in unadjusted (fold difference, 0.97 [95% CI, .94–1.00] and 0.93 [.87–.99], respectively; both *P* = .03) and adjusted models (Table 2).

**Table 2.** Relationship between markers of virus persistence and percentage of cells expressing CCR6 or CXCR3 in rectal and LN tissue

	Fold Change^a^ (95% CI) [*P* Value]					
CKR	Lymph Node Tissue			Rectal Tissue		
	Integrated HIV DNA (n = 7)	CA-US RNA (n = 7)	RNA-to-DNA Ratio (n = 7)	Integrated HIV DNA (n = 14)	CA-US RNA (n = 12)	RNA-to-DNA Ratio (n = 11)
Unadjusted						
R6^+^X3^+^	0.96 (.73–1.26) [.79]	0.70 (.44–1.09) [.11]	0.84 (.67–1.06) [.15]	0.97 (.94–1.00) [.03]^b^	0.93 (.87–.99) [.03]^b^	0.96 (.93–1.00) [.07]
R6^+^X3^−^	0.95 (.84–1.07) [.41]	0.84 (.71–1.00) [.046]^b^	0.90 (.80–1.02) [.11]	1.04 (1.01–1.09) [.02]^b^	1.08 (1.01–1.16) [.03]^b^	1.06 (1.01–1.11) [.02]^b^
R6^−^X3^+^	1.01 (.92–1.12) [.79]	1.13 (.97–1.31) [.12]	1.09 (.98–1.20) [.10]	1.04 (.92–1.17) [.57]	0.97 (.78–1.19) [.75]	1.00 (.84–1.18) [.95]
R6^−^X3^−^	1.09 (.92–1.29) [.31]	1.34 (.92–1.95) [.13]	1.06 (.89–1.26) [.50]	1.05 (.92–1.21) [.44]	1.02 (.75–1.39) [.91]	1.03 (.90–1.18) [.67]
Adjusted for current CD4^+^ T-cell count						
R6^+^X3^+^	0.98 (.80–1.19) [.82]	0.70 (.49–1.00) [.05]	ND	0.96 (.93–.99) [.02]^b^	0.90 (.85–.97) [.003]^b^	ND
R6^+^X3^−^	0.96 (.87–1.07) [.49]	0.82 (.72–.93) [.003]^b^	ND	1.05 (1.01–1.09) [.02]^b^	1.12 (1.05–1.20) [.<001]^b^	ND
R6^−^X3^+^	1.01 (.93–1.10) [.76]	1.15 (1.05–1.27) [.003]^b^	ND	1.03 (.92–1.17) [.59]	0.93 (.72–1.18) [.54]	ND
R6^−^X3^−^	1.04 (.90–1.21) [.58]	1.18 (.83–1.67) [.35]	ND	1.06 (.92–1.21) [.44]	1.02 (.74–1.42) [.90]	ND
Adjusted for nadir CD4^+^ T-cell count						
R6^+^X3^+^	1.00 (.71–1.42) [1.00]	0.64 (.36–1.13) [.12]	ND	0.96 (.94–.99) [.02]^b^	0.93 (.87–.99) [.03]^b^	ND
R6^+^X3^−^	0.93 (.77–1.13) [.47]	0.69 (.55–.88) [.002]^*b*^	ND	1.05 (1.01–1.09) [.02]^b^	1.09 (1.01–1.17) [.02]^b^	ND
R6^−^X3^+^	0.99 (.85–1.16) [.93]	1.22 (1.01–1.49) [.04]^b^	ND	1.04 (.92–1.17) [.56]	0.93 (.73–1.18) [.54]	ND
R6^−^X3^−^	1.09 (.91–1.29) [.35]	1.33 (.90–1.95) [.15]	ND	1.06 (.92–1.23) [.40]	1.01 (.73–1.40) [.96]	ND
Adjusted for current and nadir CD4^+^ T-cell count						
R6^+^X3^+^	ND	ND	ND	0.96 (.93–.99) [.005]^b^	0.91 (.85–.97) [.004]^b^	ND
R6^+^X3^−^	ND	ND	ND	1.05 (1.01–1.10) [.007]^b^	1.12 (1.05–1.20) [.001]^b^	ND
R6^−^X3^+^	ND	ND	ND	1.04 (.92–1.17) [.56]	0.89 (.69–1.15) [.38]	ND
R6^−^X3^−^	ND	ND	ND	1.07 (.92–1.25) [.37]	1.01 (.72–1.41) [.96]	ND

Markers of virus persistence ie integrated HIV DNA and cell associated (CA)-unspliced (US) HIV RNA were quantified in total CD4+ T-cells at each tissue site.

Abbreviations: CKR, chemokine receptor; ND, not determined; R6, CC56; X3, CXCR3.

^a^For each 1-unit increase in predictor (percentage of total memory CD4^+^ T cells expressing CCR6 and/or CXCR3 combinations), the fold change in HIV reservoir outcome in the same tissue is shown.

^b^Significant at *P* < .05.

Different relationships were observed in LN tissue. Integrated HIV DNA had no statistically significant relationships with frequencies of T cells expressing specific CKRs (Table 2 and Supplementary Table 4). HIV CA-US RNA had a statistically significant positive relationship with the frequency of CCR5^+^ T cells in unadjusted (fold difference, 1.15 [95% CI, 1.04–1.26]; *P* = .004) and adjusted models but an inverse relationship to the frequency of CXCR5^+^ and CCR6^+^CXCR3^−^ T cells in unadjusted (fold difference, 0.88 [95% CI, .81–.96] [*P* = .004] and .84 [.71–1.00] [*P* = .046], respectively) and adjusted models (Table 2 and Supplementary Table 4).

### Contribution of HIV-Infected T Cells Expressing CCR6 and CXCR3 to HIV Persistence in Rectal Tissue

To assess directly whether there was preferential enrichment of HIV in specific CCR6/CXCR3 T-cell subsets in rectum, a second sigmoidoscopy was performed and blood samples collected in 10 participants from the original cohort (demographic details in Supplementary Table 5). Because HIV is present in CD4^+^ and CD4^−^ T cells in GALT [40], to capture all infected cells, CD8^−^ rather than CD4^+^ memory T cells were sorted into CCR6/CXCR3 subsets (Supplementary Figure 3). Similar distributions of CCR6/CXCR3 subsets were seen in rectal tissue from these participants (Supplementary Figure 4) compared to the original cohort (Figure 2). There were some minor differences in subset distribution in blood samples from this cohort versus the original cohort, possibly owing to sorting CD8^−^ cells here rather than CD4^+^ memory cells originally (Supplementary Figure 4A and Figure 2, respectively).

**Figure 4.**
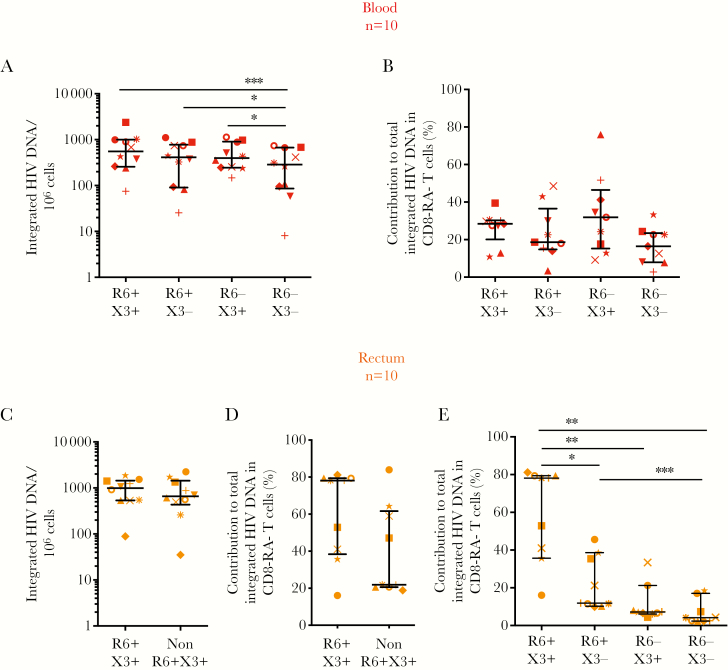
Rectal CCR6^+^ memory CD8^−^ T cells have a large contribution to the total pool of integrated human immunodeficiency virus (HIV) in memory CD8^−^ T cells. Single cells isolated from peripheral blood (*A, B*) or rectal biopsy specimens (*C–E*) from 10 people living with HIV and receiving antiretroviral therapy were sorted into CD45^+^, CD3^+^, CD45RA^−^ CD8^−^ memory T cells (CD8^−^ RA^−^ T cells) that expressed CCR6 and/or CXCR3 or neither. Integrated HIV DNA was quantified in subsets isolated from blood (*A*) or rectal tissue (*C*). The relative contribution of each subset to the total integrated HIV DNA reservoir was calculated based on the frequency of the specific T-cell subset and the level of integrated HIV DNA in either blood (*B*) or rectal tissue (*D, E*). For rectal tissue, relative contribution was measured for the 2 sorted CCR6^+^CXCR3^+^ and non-CCR6^+^CXCR3^+^ subsets (*D*) and extrapolated for the 4 CCR6/CXCR3 subsets using the frequencies of the cells together with integrated HIV DNA in the non-CCR6^+^CXCR3^+^ T-cell pool to calculate relative contribution for the individual CCR6^+^CXCR3^−^, CCR6^−^CXCR3^+^, and CCR6^−^CXCR3^−^ subsets (*E*). Data are displayed as median and interquartile range, and each symbol represents an individual donor. *P* values <.05 (Wilcoxon matched-pairs signed rank test) are shown. Abbreviations: Non-R6^+^X3^+^, pooled cell fraction containing R6^+^X3^−^, R6^−^X3^+^ and R6^−^X3^−^ subsets together; R6, CCR6; X3, CXCR3. *P < .05, **P < .01, ***P < .005.

In blood, integrated HIV DNA in CCR6^+^CXCR3^+^, CCR6^+^CXCR3^−^, and CCR6^−^CXCR3^+^ subsets compared to CCR6^−^CXCR3^−^ memory T cells was substantially higher (median fold increase, 1.93 [*P* = .002], 1.44 [*P* = .049], and 1.38 [*P* = .03], respectively) (Figure 4A). However, when the frequency of each cell subset was accounted for, there was no statistically significant difference between the relative contributions of the different subsets to the total pool of cells harboring integrated HIV DNA in blood (Figure 4B).

In rectal tissue, owing to low total cell numbers, only CCR6^+^CXCR3^+^ T cells and the remaining T-cell pool (non-CCR6^+^CXCR3^+^) were sorted. There was no statistically significant enrichment of integrated HIV DNA in CCR6^+^CXCR3^+^ versus the non-CCR6^+^CXCR3^+^ T-cell pool (Figure 4C). The median contributions of these 2 subsets to the total integrated DNA reservoir in rectal tissue were 78.1% for CCR6^+^CXCR3^+^ T cells and 21.9% for the non-CCR6^+^CXCR3^+^ T-cell pool, but these differences were not statistically significant (*P* = .36) (Figure 4D).

Given the mixed population in the non-CCR6^+^CXCR3^+^ T-cell pool, we next calculated the relative contribution of each subset to the total integrated DNA reservoir in rectal tissue, using the frequency of each subset and the HIV DNA concentration in CCR6^+^CXCR3^+^ or non-CCR6^+^CXCR3^+^ cells (as an estimate of DNA concentration in CCR6^+^CXCR3^−^, CCR6^−^CXCR3^+^, and CCR6^−^CXCR3^−^ subsets). The CCR6^+^CXCR3^+^ subset had a significantly greater contribution to the total integrated HIV DNA reservoir in rectal tissue compared with the remaining 3 subsets (Figure 4E), owing to the high frequency of CCR6^+^CXCR3^+^ cells in rectal tissue (Supplementary Figure 4*B*). CCR6^+^CXCR3^−^ T cells also had a significantly greater contribution than CCR6^−^CXCR3^−^ T cells to the integrated HIV DNA reservoir (Figure 4E). Therefore, although rectal CCR6^+^CXCR3^+^ cells were not preferentially infected, infected CCR6^+^ cells accounted for nearly all integrated HIV DNA detected in rectal tissue (median, 89.7%; IQR, 68.3%–90.5%).

By comparison, in blood, CCR6^+^CXCR3^+^ T cells were less frequent (Supplementary Figure 4), and CCR6^+^CXCR3^+^ together with CCR6^+^CXCR3^−^ T cells had a combined median 53.1% (IQR, 43.9%–57.7%) contribution to the total integrated HIV DNA reservoir in blood. The contribution of all CCR6^+^ T cells to the total pool of HIV-infected cells in blood compared with rectal tissue was significantly lower (*P* = .02; Wilcoxon signed rank test). Hence both CCR6^+^ and CCR6^−^ T cells had an important contribution to the HIV reservoir in blood, whereas CCR6^+^ T cells accounted for most of the HIV reservoir in rectal tissue, as measured by integrated HIV DNA.

### Enrichment of Rectal Tissue in CCL20 mRNA and LN Tissue in CCL19, CCL21, and Other Chemokine mRNA CCL20 mRNA in Rectal Tissue and CCL19 and CCL21 in LN Tissue

**Figure 5.**
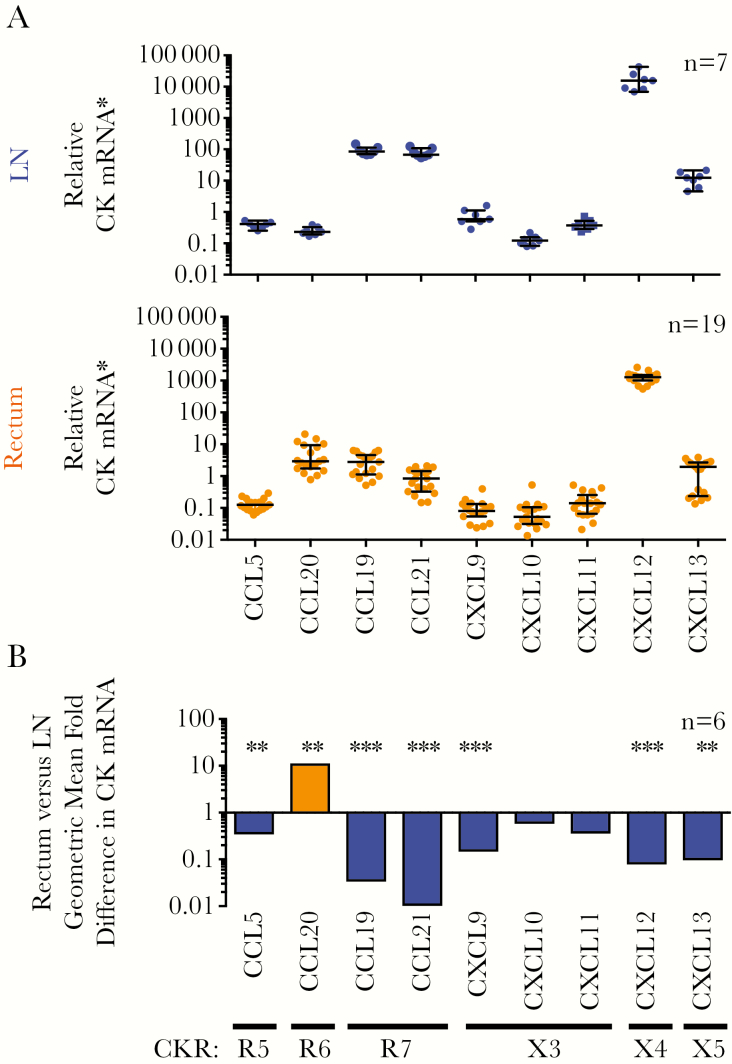
Rectal tissue (upper panel) is enriched in CCL20 messenger RNA (mRNA), and lymph node (LN) tissue (middle panel) is enriched in CCL19, CCL21, and other chemokine mRNA. *A,* Total RNA was extracted from rectal pinch biopsy specimens or LN biopsy slices collected from people living with human immunodeficiency virus (HIV) and receiving antiretroviral therapy. Next, 100-ng aliquots of total RNA were analyzed for chemokine mRNA or 18S ribosomal RNA (rRNA) expression using quantitative reverse-transcription polymerase chain reaction. Chemokine RNA levels were normalized to cellular 18S rRNA, and relative chemokine mRNA was calculated as the fold difference in samples relative to a calibrator (untreated peripheral blood mononuclear cells) from a healthy donor for most chemokines or liver tissue for CCL21 mRNA). Data are displayed as median and interquartile range, and each dot represents an individual donor. *B,* For participants who donated both rectal and LN samples (n = 6), the geometric mean fold difference in chemokine mRNA for rectal tissue compared with LN tissue is shown for each chemokine (*vertical labeling*). Chemokine receptors (CKRs) that bind chemokines encoded by the chemokine mRNA are also shown. Relative chemokine mRNA levels in rectal and LN tissue were log-transformed and compared using a paired *t* test (Supplementary Table 6). *P < .01; †*P* < .001. Abbreviations: R5, CCR5; R6, CCR6; R7, CCR7: X3, CXCR3; X4, CXCR4; X5, CXCR5.

Next, the expression of relevant chemokine RNAs was quantified in tissues. All chemokines were detected in LN and rectal tissues (Figure 5A). CXCL12 (CXCR4 ligand) had the highest RNA expression of all chemokines measured in both tissues (Figure 5A). As expected, there was higher RNA expression of CCL20 (CCR6 ligand) in rectal tissue and CCL19 plus CCL21 (CCR7 ligands) in LN tissue, consistent with CCR6 or CCR7 expressing cells homing to these 2 tissues, respectively [14, 15]. For 6 participants with paired rectal and LN tissue samples, most chemokines had significantly higher expression in LN tissue, except CCL20, which was 11.4-fold higher in rectal than in LN tissue (Figure 5B and Supplementary Table 6).

### Relation Between HIV Persistence During ART and Chemokine Expression in LN Tissue

HIV persistence markers were compared with chemokine expression (Supplementary Table 7). In LN tissue, there were statistically significant inverse associations between integrated HIV DNA and both CCL19 and CCL5 mRNA (*P* = .002 and *P* = .02, respectively) and between HIV CA-US RNA and both CCL19 and CCL21 mRNA (*P* = .001 and *P* = .02, respectively) in unadjusted and most adjusted models. HIV CA-US RNA had a statistically significant positive relationship to CXCL13 mRNA in unadjusted (*P* = .03) and nadir CD4^+^ T-cell count–adjusted models (Supplementary Table 7). In rectal tissue, chemokine mRNAs had no statistically significant relationships with any markers of HIV persistence.

## DISCUSSION

This study is unique in systematically evaluating the frequency of T-cell subsets in blood and in LN and rectal tissue based on CKR expression in PLWH on suppressive ART. We then used these data to calculate the overall contribution of specific CKR-expressing cell types to HIV persistence during ART. The very high frequency of CCR6^+^ T cells in rectal tissue identified a distinct subset of T cells that are a major contributor to HIV persistence during ART. Furthermore, the different relationships between HIV persistence and T-cell subsets and chemokines in rectal and LN tissue suggests that different tissue-specific strategies may be required to eliminate HIV persistence.

We made a number of key observations. The frequency of HIV-infected cells was higher in CD4^+^ T cells from rectal tissue versus blood, as our group reported elsewhere [10]. Rectal tissue was highly enriched for CCR6^+^CXCR3^+^ T cells and the CCR6 ligand, CCL20. Although integrated HIV DNA was not substantially enriched in CCR6^+^CXCR3^+^ T cells from rectal tissue versus all other cells, infected CCR6^+^ T cells accounted for nearly all infected cells in the rectum. Conversely in LN tissue, there was a statistically significant relationship between HIV persistence markers and chemokines that bind to CCR7, CCR5, and CXCR5. Therefore, CKR-expressing cells and chemokines had a different relationship with HIV persistence in rectal and LN tissue among those receiving effective ART.

The enrichment of HIV DNA and RNA in rectal CD4^+^ T cells compared with blood (Supplementary Table 1) is consistent with findings of prior studies in human [7, 8, 10] and nonhuman primates [41]. Although our study found more HIV CA-US RNA in rectal and LN tissue than in blood, the ratio of HIV US RNA to integrated DNA was similar across all 3 anatomic sites (Table 1 and Supplementary Table 1). This suggests there were no site-specific effects on basal levels of HIV RNA transcription. However, given that polymerase chain reaction–based assays can only assess the average value across all cells in the tissue, basal HIV transcription may differ within tissue, as reported for B-cell follicles in LN tissue [9, 42].

We observed both identical and distinct HIV *env* sequences in blood, LN or rectal tissue within an individual. There was no evidence for HIV compartmentalization between blood and tissue, in agreement with findings of some studies [7, 43] but not others [44, 45]. Our findings may reflect the limited number of sequences or participants analyzed and/or inclusion of participants treated during chronic infection, where compartmentalization of proviral sequences in gut and blood might be less pronounced than described for participants treated during infection [46]. However, because HIV sequences from tissue were not always represented in blood, and CD4^+^ T cells from rectal tissue compared to blood have a greater block to HIV transcription initiation [47], ongoing work is still required to better define, quantify, and target infected cells that persist during ART in tissue sites.

The high frequency of CXCR3^+^CCR6^+^ T cells (Figure 2) and CCL20 mRNA expression in rectal tissue (Figure 5) is consistent with CCR6^+^ T cells homing to gastrointestinal tissue [15]. CXCR3^+^ T-cell subsets also coexpressed more CCR5 than their CXCR3^−^ counterparts in all anatomic sites (Figure 2C), possibly reflecting the role of CXCR3 and CCR5 as inflammatory CKRs for migration to inflammatory sites [13, 16]. In rectal tissue, the high proportion of CCR6^+^CXCR3^+^ T cells coexpressing the CCR5 HIV coreceptor (Figure 2B and 2C) may allow preferential productive infection of this subset. However, and unexpectedly, CCR6^+^CXCR3^+^ memory T cells from rectal tissue were not substantially enriched in integrated HIV DNA compared other T-cell subsets combined (Figure 4C). In contrast, blood CCR6^+^CXCR3^+^, CCR6^+^CXCR3^−^ and CCR6^−^CXCR3^+^ T cells were enriched in integrated HIV DNA compared with CCR6^−^CXCR3^−^ cells (Figure 4A) [28].

Our findings in relation to HIV in rectal tissue could be explained by the comparison of CCR6^+^CXCR3^+^ cells with a pooled fraction still containing substantial levels of CCR6^+^CXCR3^−^ T cells (Supplementary Figure 4*B*), which may have a high frequency of infected cells. Supporting this interpretation, we found a positive relationship between HIV DNA or RNA with CCR6^+^CXCR3^−^ T-cell frequency in rectal tissue (Table 2), and others report that CCR6^+^ versus CCR6^−^ T cells from the colon are enriched in HIV Gag DNA in PLWH receiving ART [29]. Alternatively, these findings may be explained by a trafficking defect of infected CCR6^+^CXCR3^+^ T cells into or out of rectal tissue, causing these infected cells to accumulate in blood [48, 49].

The relationships between CKRs, chemokines, and HIV in LN tissue differed from those in rectal tissue. In LN tissue, HIV CA-US RNA was positively associated with CXCL13 mRNA and inversely associated with CXCR5^+^ CD4^+^ T-cell frequency, but those relationships were weaker or in the opposite direction in rectal tissue (Supplementary Tables 4 and 7). CXCL13 is the ligand for CXCR5, and HIV RNA is enriched in CXCR5^+^PD1^hi^ CD4^+^ T cells in LN tissue [9]. Because CXCR5 is expressed on CD4^+^ and CD8^+^ T cells, it is possible that elevated CXCL13 recruits more CXCR5^+^ CD8^+^ T cells into LN tissue, enhancing the depletion of HIV RNA expressing cells like CXCR5^+^ CD4^+^ T cells, resulting in an inverse association of CA-US HIV RNA with the frequency of CXCR5^+^ CD4^+^ T cells.

In LN tissue, integrated HIV DNA and CA-US RNA were inversely associated with CCL19 and CCL21 mRNA (Supplementary Table 7). We were unfortunately not able to measure CCR7-expressing cells. However, because CCL19 and CCL21 recruit CCR7^+^ T cells to LNs [14], enhanced recruitment of CCR7^+^ CD8^+^ T cells by these chemokines might contribute to the death of cells expressing HIV RNA. Alternatively, CCL19/CCL21-enhanced recruitment of uninfected CD4^+^ T cells to LNs might also dilute the pool of HIV-infected cells, causing an inverse association of CCL19 and CCL21 with HIV DNA and RNA.

Although the key contribution of CCR6^+^ T cells to HIV persistence in rectal tissue is consistent with a previous study [29], neither study examined whether rectal CCR6/CXCR3 subsets preferentially harbor intact or inducible, replication-competent HIV. This should be investigated in future. Other limitations of our study include the measurement of chemokine mRNA in tissue rather than protein expression. Any changes in translation or posttranslational modifications of chemokine proteins could yield different findings to mRNA expression. Staining tissue for chemokine protein and HIV DNA/RNA in future studies could provide insights into relationships between chemokines and infected cells. Moreover, given that this was a cross-sectional study, causation could not be determined. Of note, because we and others find HIV enriched in T cells that express PD1 and other immune checkpoint markers during ART [9, 34, 50], assessing these immune checkpoint markers in relation to CKR expression is also of further interest.

In conclusion, we show that HIV-infected CCR6^+^ CD4^+^ T cells account for nearly all HIV-infected cells in rectal tissue PLWH but not in blood. Therefore, interventions targeting CCR6^+^ T cells may be particularly effective at reducing HIV persistence during ART in gastrointestinal tissue and should be considered. The different relationships between HIV and CKR or chemokines in LN and rectal tissue indicate that different strategies may be needed to eliminate HIV persistence at different tissue sites. Furthermore, biomarkers for HIV persistence in blood may not provide an accurate assessment of the total HIV-infected cell pool in tissue.

## Supplementary Data

Supplementary materials are available at *The Journal of Infectious Diseases* online. Consisting of data provided by the authors to benefit the reader, the posted materials are not copyedited and are the sole responsibility of the authors, so questions or comments should be addressed to the corresponding author.

jiz509_suppl_Supplmentary_Figure_1

jiz509_suppl_Supplmentary_Figure_2

jiz509_suppl_Supplmentary_Figure_3

jiz509_suppl_Supplmentary_Figure_4

jiz509_suppl_Supplmentary_Table_1

jiz509_suppl_Supplmentary_Table_2

jiz509_suppl_Supplmentary_Table_3

jiz509_suppl_Supplmentary_Table_4

jiz509_suppl_Supplmentary_Table_5

jiz509_suppl_Supplmentary_Table_6

jiz509_suppl_Supplmentary_Table_7

jiz509_suppl_Supplmentary_text

## Notes

***Acknowledgments.*** We are most grateful to the study participants for their generous donation of samples. We also thank the University of California San Francisco, Clinical Core Laboratory for recruiting patients and collecting and processing specimens, and the University of California, San Francisco, and the Vaccine and Gene Therapy Institute of Florida Flow Cores for cell sorting.

***Author contributions***. J. L. A., N. C., E. S., J. M. M., M. R., P. U. C., S. G. D., and S. R. L. conceived and designed the experiments. C. L. E., R. H., F. M. H., and M. S. recruited participants and collected and processed initial specimens. J. L. A., G. K., R. F., A. S., N .C., E. S., J. M. M., C. T., M. G., and M. S. performed experiments. J. L. A., G. K., R. F., A. S., E. S., J. M., M. R., and M. G. analyzed the data. P. B. advised on and J. L. A. and W. H. performed statistical analyses. J. L. A. and S. R. L. wrote the manuscript. All authors reviewed and approved of the final manuscript.

***Financial support.*** This work was supported by the National Institutes of Health Delaney AIDS Research Enterprise Collaboratory (grants U19 A1096109 and UM1AI126611), the National Health and Medical Research Council of Australia (program grant and practitioner fellowship to S. R. L. and postgraduate scholarships 579719 to G. K. and 1114265 to M. C. P.), and the Royal Australasian College of Physicians (CRB Blackburn Scholarship to M. C. P.).

***Potential conflicts of interest.*** J. L. A. and S. R. L. have research collaborations with Merck Sciences unrelated to the current work. N. C. has research collaborations with Merck and EMD Serono unrelated to the current work. S. G. D. has received grant support from Gilead, Merck, and ViiV, has consulted for AbbVie, Janssen, and Shionogi, and is a member of the scientific advisory boards for BryoLogyx and Enochian Biosciences. S. R. L. has received investigator-initiated, industry-funded research support from Merck Sciences, Gilead Sciences, and ViiV, and provision of reagents from Infinity Pharmaceuticals, Merck Sciences, and Bristol-Myers Squibb for investigator-initiated research. All other authors report no potential conflicts. All authors have submitted the ICMJE Form for Disclosure of Potential Conflicts of Interest. Conflicts that the editors consider relevant to the content of the manuscript have been disclosed.

Presented in part: AIDS2014 20th International AIDS Conference, Melbourne, Australia, 20-25 July 2014; Conference on Retroviruses and Opportunistic Infections 13-16 February 2017, Seattle, Washington; Australian Centre for HIV and Hepatitis Virology Research 11th Annual Scientific Workshop, Hunter Valley, Australia, 9-11 December 2015 (abstract 30400); Australian Centre for HIV and Hepatitis Virology Research 12th Annual Scientific Workshop, Barossa Valley, Australia, 7-9 June 2017 (abstract 4); Australasian HIV and AIDS 2016 Conference, Adelaide, Australia, 16-18 November 2016; and Australasian HIV and AIDS 2018 Conference, Sydney, Australia, 24-26 September 2018 (abstract 471).
