# Supplementary material for: Human Immunodeficiency Virus (HIV)–Infected CCR6+ Rectal CD4+ T Cells and HIV Persistence On Antiretroviral Therapy
Source: J Infect Dis. 2019 Dec 4;221(5):744–55. doi: 10.1093/infdis/jiz509 (PMC7026892; doi:10.1093/infdis/jiz509)

Supplementary Figure 1

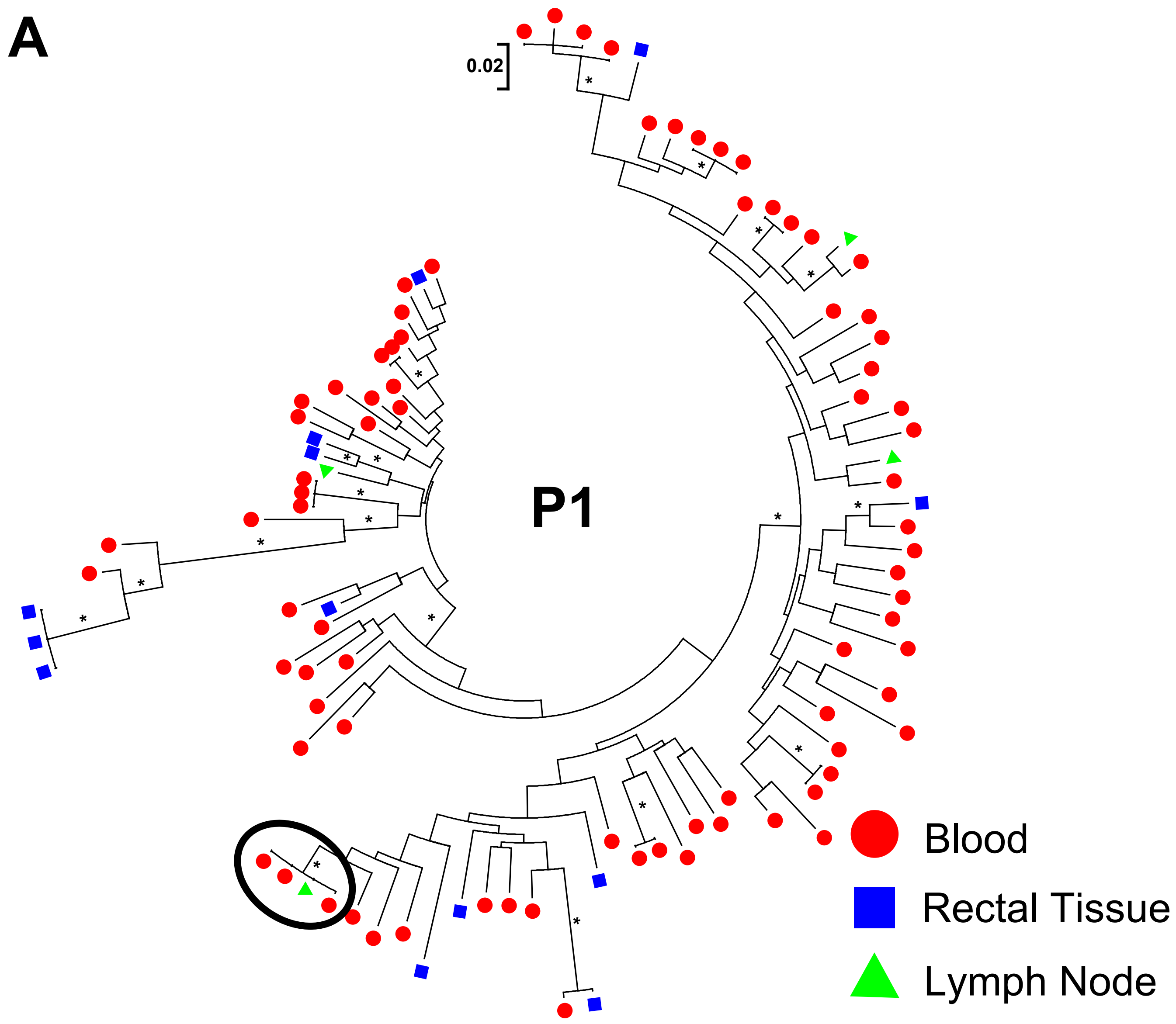

Supplementary Figure 1: Phylogenetic analysis of HIV *env* sequences from peripheral blood, LN and rectal tissue CD4+ T cells from people living with HIV on ART. Maximum likelihood trees for HIV *env* sequences from peripheral blood (*red circle*), LN (*green triangle*) or rectal tissue (*blue square*) CD4+ T-cells from five participants on ART (A-E). Hypermutated sequences were removed from analyses. Scale bars show numbers of nucleotide substitutions per site. Asterisks denote nodes supported by >75% bootstraps. Ovals mark identical sequences in blood and tissue.

Supplementary Figure 1

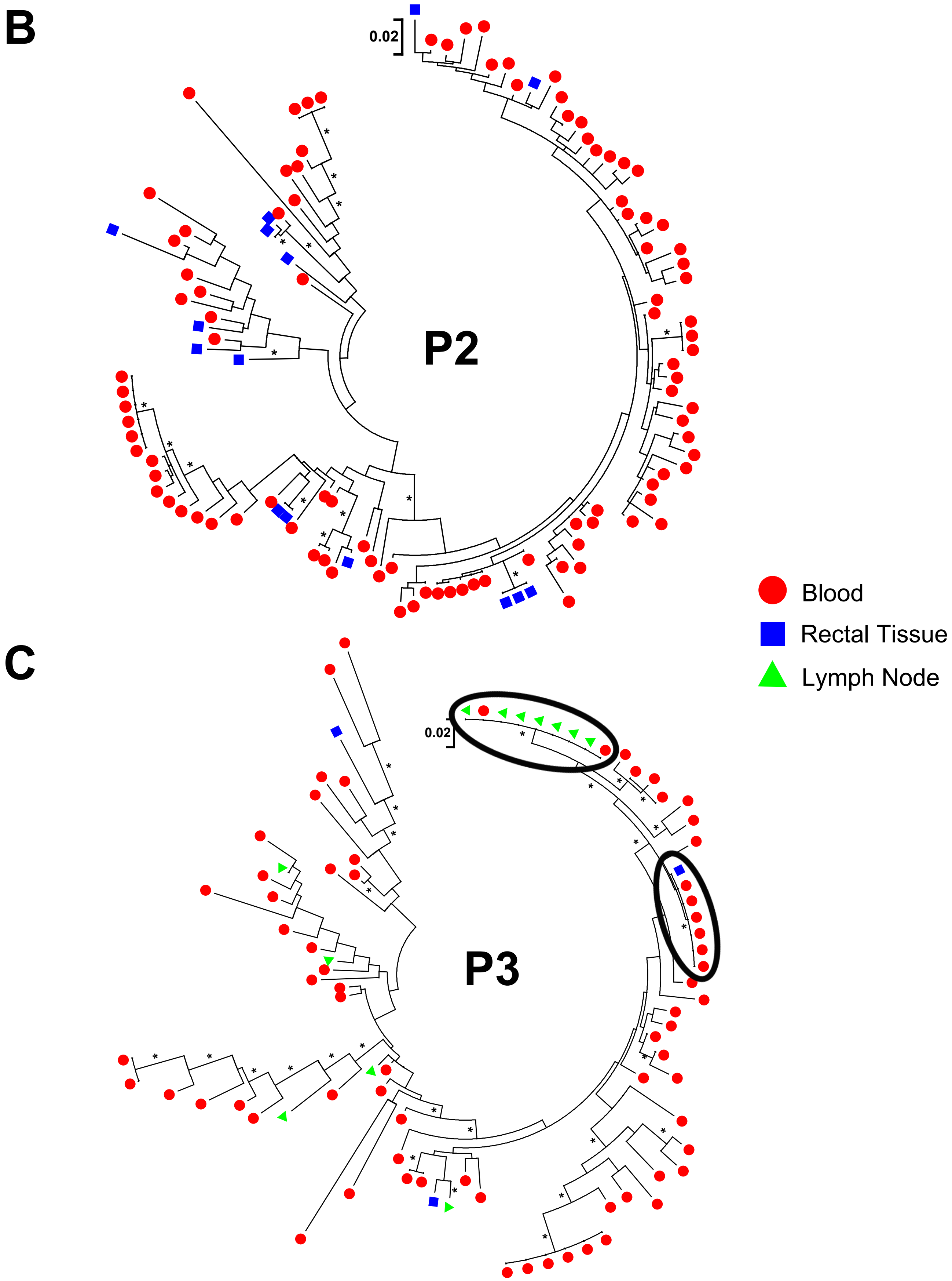

Supplementary Figure 1

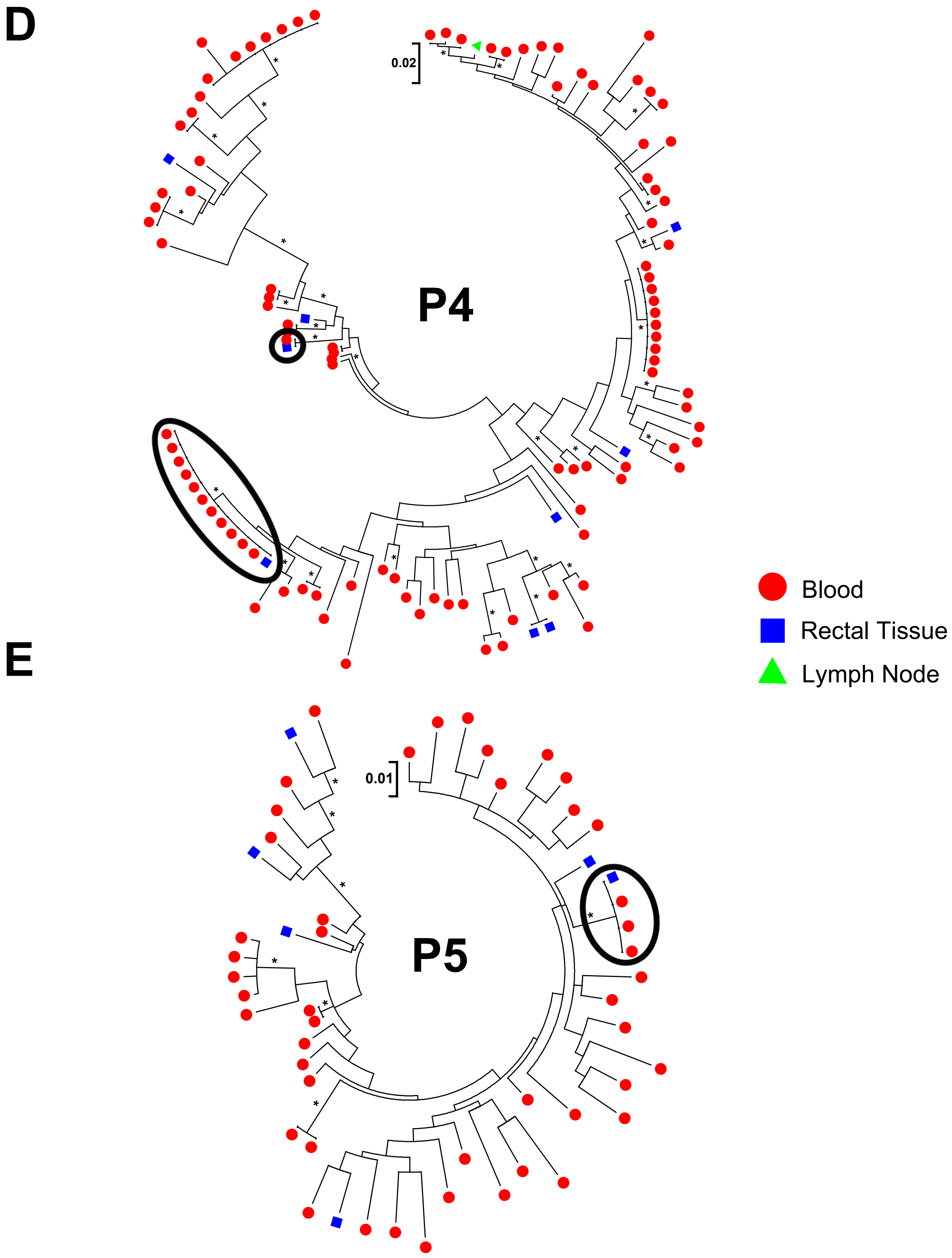

Supplement: jiz509_suppl_Supplmentary_Figure_1 [file jiz509_suppl_supplmentary_figure_1.pdf]
