# Supplementary material for: Human Immunodeficiency Virus (HIV)–Infected CCR6+ Rectal CD4+ T Cells and HIV Persistence On Antiretroviral Therapy
Source: J Infect Dis. 2019 Dec 4;221(5):744–55. doi: 10.1093/infdis/jiz509 (PMC7026892; doi:10.1093/infdis/jiz509)
Supplement: jiz509_suppl_Supplmentary_Figure_2 [file jiz509_suppl_supplmentary_figure_2.pdf]

Supplementary Figure 2

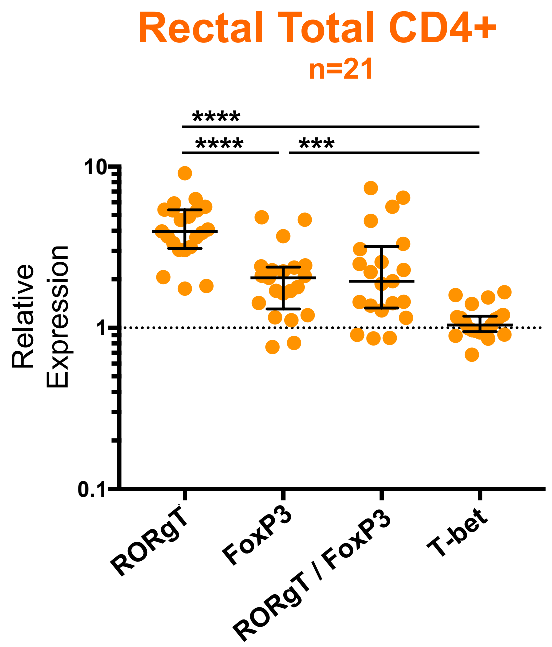

Supplementary Figure 2: Relative expression of RORγT, FoxP3 and T-bet transcription factors in rectal total CD4+ T-cells. The relative expression of T-cell transcription factor mRNAs was assessed in total CD4+ T-cells from rectal tissue from people living with HIV on ART (n=21) using RT-qPCR. Transcription factors include: RORγT that marks Th17 cells, FoxP3 that marks Treg cells, ratio of RORγT:FoxP3 that marks the abundance of Th17 to Treg cells, and T-bet that marks Th1 cells. The relative expression of mRNA in rectal samples relative to levels in a calibrator sample, here being RNA from PBMC collected from a single healthy donor (dotted line at 1) is shown, with the median bar and interquartile range indicated (*black bars*). Differences between transcription factor levels was assessed using the Wilcoxon matched-pairs signed rank test with P values <0.05 shown as asterisks (*** P <0.001, **** P <0.0001).
