# Supplementary material for: Human Immunodeficiency Virus (HIV)–Infected CCR6+ Rectal CD4+ T Cells and HIV Persistence On Antiretroviral Therapy
Source: J Infect Dis. 2019 Dec 4;221(5):744–55. doi: 10.1093/infdis/jiz509 (PMC7026892; doi:10.1093/infdis/jiz509)
Supplement: jiz509_suppl_Supplmentary_Figure_3 [file jiz509_suppl_supplmentary_figure_3.pdf]

Supplementary Figure 3

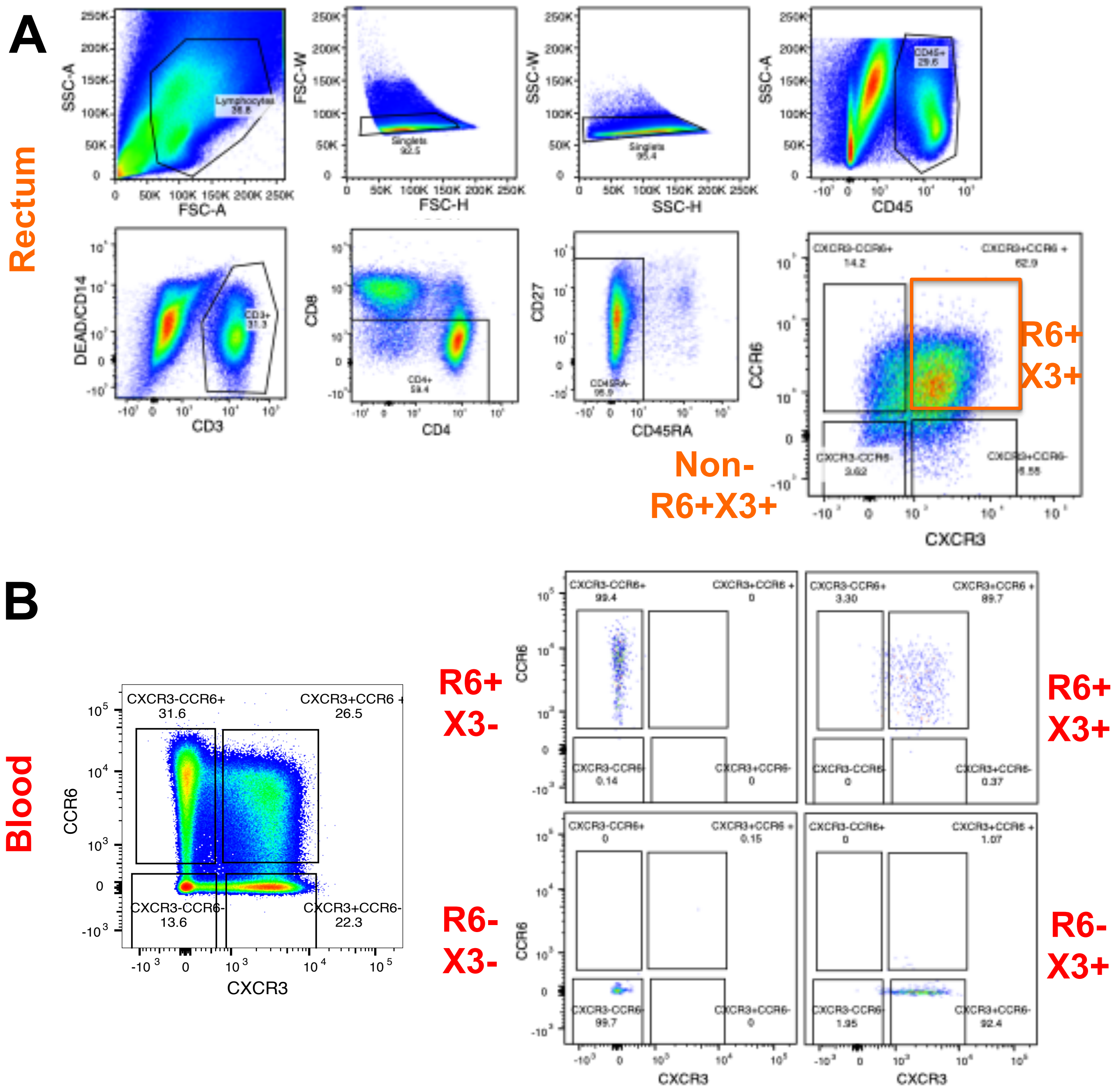

Supplementary Figure 3: Representative gating strategy of CCR6/CXCR3 T-cell subsets sorted from rectal tissue (A) or peripheral blood (B) from a participant on ART. The full gating strategy preceding CCR6 (R6) versus CXCR3 (X3) shown for rectal tissue (A) was also used for blood cells (B, *data not shown*). Purity checks of blood sorted R6/X3 subsets were performed (B, *right panel*). Due to limited cells, rectal T-cells were sorted into R6+X3+ cells and a pooled subset of the other 3 cell types (non-R6+X3+) and purity checks not performed.
