# Supplementary material for: Human Immunodeficiency Virus (HIV)–Infected CCR6+ Rectal CD4+ T Cells and HIV Persistence On Antiretroviral Therapy
Source: J Infect Dis. 2019 Dec 4;221(5):744–55. doi: 10.1093/infdis/jiz509 (PMC7026892; doi:10.1093/infdis/jiz509)
Supplement: jiz509_suppl_Supplmentary_Figure_4 [file jiz509_suppl_supplmentary_figure_4.pdf]

Supplementary Figure 4

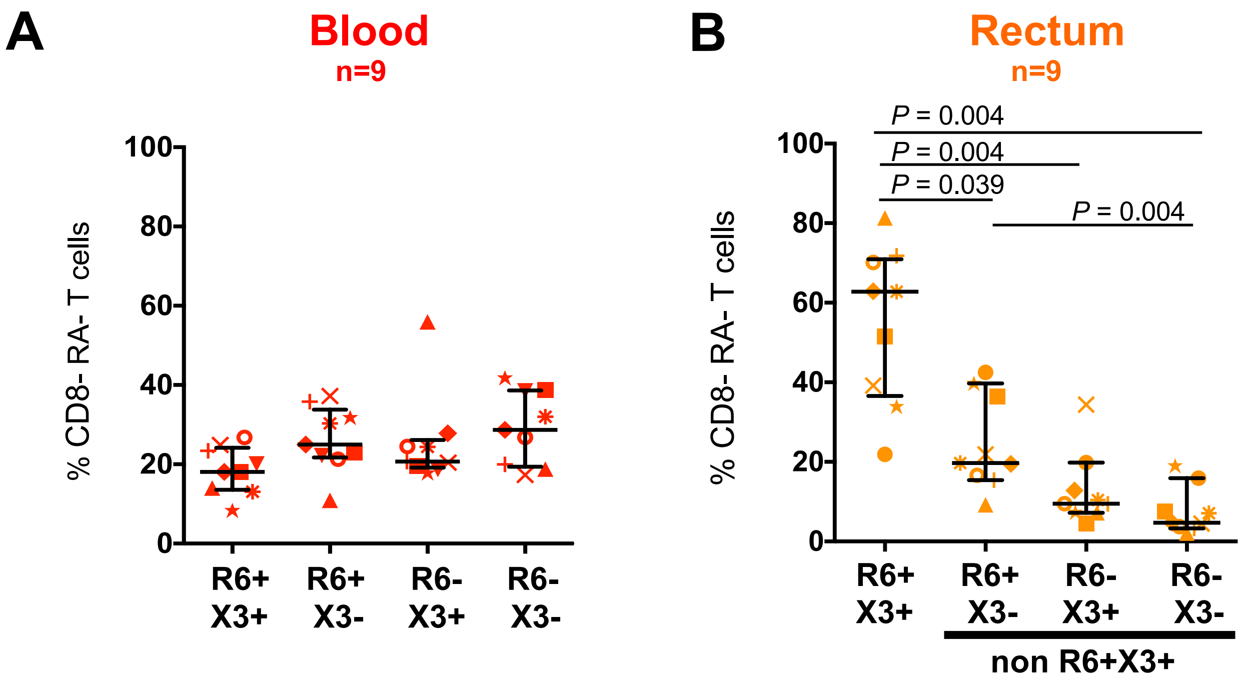

Supplementary Figure 4: Rectal tissue is enriched in memory CD8- T-cells that co-express CCR6+CXCR3+. Single cell suspensions isolated from peripheral blood (A) or 30 rectal pinch biopsies (B) from 10 people living with HIV on ART were sorted into CD45+, CD3+, CD45RA- CD8- memory T-cells (CD8- RA- T-cells) that expressed CCR6 and/or CXCR3 or neither. In blood, relatively even frequencies of the four CCR6/CXCR3 subsets (A) enabled all 4 subsets to be sorted for HIV integrated DNA analysis (Figure 4). Conversely in rectal tissue, the high proportion of CCR6+CXCR3+ cells but lower frequency of the remaining three subsets (B) only allowed the CCR6+CXCR3+ subset and a pooled remaining cell subset consisting of CCR6+CXCR3-, CCR6-CXCR3- and CCR6-CXCR3- cells termed the “non R6+X3+” subset to be sorted for HIV integrated DNA analysis (Figure 4). Medians and interquartile ranges (*black bars*) plus *P* values <0.05 (Wilcoxon matched-pairs signed rank test) are shown. Each symbol is a different donor. Data for 9 (n=9) of 10 donors sorted is shown due to issues with flow data files for 1 donor resulting in exclusion from analysis for blood and rectal tissue. R6, CCR6; X3, CXCR3; non R6+X3+, pooled fraction of R6+X3-, R6-X3+ and R6-X3- subsets together.
