# Supplementary material for: Human Immunodeficiency Virus (HIV)–Infected CCR6+ Rectal CD4+ T Cells and HIV Persistence On Antiretroviral Therapy
Source: J Infect Dis. 2019 Dec 4;221(5):744–55. doi: 10.1093/infdis/jiz509 (PMC7026892; doi:10.1093/infdis/jiz509)
Supplement: jiz509_suppl_Supplmentary_Table_1 [file jiz509_suppl_supplmentary_table_1.docx]

**Supplementary Table 1:** Comparison of HIV persistence in total CD4+ T cells between peripheral blood, lymph node (LN) and rectal tissue from people living with HIV (PLWH) on ART using negative binomial regression models.

|  | **HIV Reservoir** | | |
| --- | --- | --- | --- |
|  | **iDNA^a^**  **copies/10^6^ cells** | **CA-US RNA^b^**  **copies/10^6^ cells** | **USRNA:iDNA**  **ratio** |
| **Rectum**^c^ | 1263  (413.5 - 1942)  *n=19* | 69.4  (18.3 - 301.3)  *n=16* | 0.041  (0.029 - 0.201)  *n=13* |
| **LN**^c^ | 449.7  (217.1 - 1850)  *n=7* | 75.3  (13.9 - 155.8)  *n=7* | 0.086  (0.041 - 0.328)  *n=7* |
| **Blood**^c^ | 338.8  (153.4 - 700.9)  *n=48* | 22.5  (11.8 - 38.6)  *n=44* | 0.065  (0.037 - 0.122)  *n=43* |
|  | **Fold difference (95% CI, *p-value*)** | | |
| **Rectum / Blood**^d^ | **3.91**  **(2.45-6.23, *<0.001*)** | **4.57**  **(2.56-8.15, *<0.001*)** | 1.5  (0.75-3.03, *0.260*) |
| **LN / Blood**^d^ | 1.61  (0.83-3.12, *0.160*) | **3.66**  **(1.73-7.75, *<0.001*)** | 2.10  (0.81-5.46, *0.130*) |
| **Rectum / LN**^d^ | **2.42**  **(1.19-4.92, *0.014*)** | 1.25  (0.55-2.87, *0.600*) | 0.72  (0.25-2.08, *0.540*) |

^a^ iDNA, integrated DNA

^b^ CA-US RNA, cell associated unspliced HIV RNA.

^c^ Median (interquartile range) plus number of participants (n) analysed to measure HIV persistence in total CD4+ T-cells from blood, lymph node (LN) or rectum.

^d^ Fold difference (95% confidence interval, p-value) in HIV persistence markers determined using negative binomial regression.
