# Supplementary material for: Human Immunodeficiency Virus (HIV)–Infected CCR6+ Rectal CD4+ T Cells and HIV Persistence On Antiretroviral Therapy
Source: J Infect Dis. 2019 Dec 4;221(5):744–55. doi: 10.1093/infdis/jiz509 (PMC7026892; doi:10.1093/infdis/jiz509)
Supplement: jiz509_suppl_Supplmentary_Table_2 [file jiz509_suppl_supplmentary_table_2.docx]

**Supplementary Table 2:** Spearman r correlation of HIV reservoir measures in total CD4+ T cells from peripheral blood and/or lymph node (LN) and/or rectal tissue compartments from people living with HIV on ART.

| **HIV Reservoir** | **Tissue 1** | **Tissue 2** | ***n*** | **Spearman r** | **p-value** |
| --- | --- | --- | --- | --- | --- |
| **Integrated DNA** | Rectum | Blood | 19 | 0.400 | 0.090 |
|  | LN | Blood | 7 | 0.214 | 0.662 |
|  | Rectum | LN | 6 | 0.486 | 0.356 |
| **CA-US RNA** | Rectum | Blood | 14 | 0.240 | 0.409 |
|  | LN | Blood | 6 | 0.714 | 0.136 |
|  | Rectum | LN | 6 | 0.429 | 0.419 |
| **CA-US RNA / Integrated DNA** | Rectum | Blood | 11 | -0.118 | 0.735 |
|  | LN | Blood | 6 | -0.029 | >0.999 |
|  | Rectum | LN | 5 | 0.300 | 0.683 |
