# Supplementary material for: Human Immunodeficiency Virus (HIV)–Infected CCR6+ Rectal CD4+ T Cells and HIV Persistence On Antiretroviral Therapy
Source: J Infect Dis. 2019 Dec 4;221(5):744–55. doi: 10.1093/infdis/jiz509 (PMC7026892; doi:10.1093/infdis/jiz509)
Supplement: jiz509_suppl_Supplmentary_Table_3 [file jiz509_suppl_supplmentary_table_3.docx]

**Supplementary Table 3:** Compartmentalization tests of HIV *env* sequences in total CD4+ T cells from peripheral blood versus

a) rectal tissue, b) LN tissue, or c) both rectal and LN tissue, from five people living with HIV on ART.

| **Comparison of anatomical compartments** | **Participant ID^a^** | **Simmonds AI^b^** | | **Wright’s MPS^c^** | | **Compartmentalization** |
| --- | --- | --- | --- | --- | --- | --- |
|  |  | **AI Value** | **Bootstrap Value** | **K_ST_ Value** | **p-value** |  |
| **A. Blood vs**  **Rectal Tissue** | P1 | **0.578** | **0.997** | **0.051** | **0.002** | NO |
|  | P2 | **0.534** | **0.998** | 0.024 | 0.069 | NO |
|  | P4 | 0.704 | 0.875 | 0.005 | 0.245 | NO |
|  | P5 | 0.967 | 0.380 | 0.024 | 0.090 | NO |
| **B. Blood vs**  **LN Tissue** | P1  P3 | 0.887 | 0.494 | -0.004 | 0.679 | NO |
|  |  | **0.488** | **0.998** | -0.012 | 0.755 | NO |
| **C. Blood vs**  **Tissues combined** | P1  P3  P4 | **0.723** | **0.954** | **0.043** | **0.006** | NO |
|  |  | **0.517** | **0.994** | -0.010 | 0.708 | NO |
|  |  | 0.697 | 0.908 | 0.003 | 0.318 | NO |

^a^ P, participant.

^b^ Simmonds Association Index. *Results interpretation:* AI value near 0 with a bootstrap value >0.95 was considered significant evidence for compartmentalization.

^c^ Wright’s Measure of Population Subdivision. *Results interpretation:* K_ST_ value near 1 with a p-value <0.01 was considered significant evidence for compartmentalization.
