# Supplementary material for: Human Immunodeficiency Virus (HIV)–Infected CCR6+ Rectal CD4+ T Cells and HIV Persistence On Antiretroviral Therapy
Source: J Infect Dis. 2019 Dec 4;221(5):744–55. doi: 10.1093/infdis/jiz509 (PMC7026892; doi:10.1093/infdis/jiz509)
Supplement: jiz509_suppl_Supplmentary_Table_4 [file jiz509_suppl_supplmentary_table_4.docx]

**Supplementary Table 4:** Relationship of HIV reservoir in total CD4+ T cells to the percentage of single chemokine receptor expressing total memory CD4+ T cells in LN and rectal tissues from people living with HIV on ART using negative binomial regression.

| **CKR^1^** | **LN^2^** | | | | **RECTUM** | | | |
| --- | --- | --- | --- | --- | --- | --- | --- | --- |
|  | **Int DNA^3^**  **n=7** | **CA-US RNA^3^**  **n=7** | **CA-US RNA: intDNA^3^**  **n=7** | | **Int DNA^3^**  **n=15** | **CA-US RNA^3^**  **n=12** | **CA-US RNA: intDNA^3^**  **n=11** | |
| **Unadjusted** | | | | | | | | |
| **CCR5** | 1.08 (0.99 to 1.17) *p=0.07* | **1.15 (1.04 to 1.26) *p=0.0041*** | | 1.08 (0.99 to 1.17)  *p=0.07* | 1.01 (0.97 to 1.05) *p=0.78* | 1.01 (0.94 to 1.09) *p=0.80* | | 0.98 (0.94 to 1.03) *p=0.49* |
| **CCR6** | 0.97 (0.89 to 1.06) *p=0.52* | 0.89 (0.78 to 1.00) *p=0.054* | | 0.93 (0.86 to 1.02)  *p=0.11* | 0.97 (0.90 to 1.05) *p=0.42* | 1.02 (0.87 to 1.18) *p=0.85* | | 0.99 (0.91 to 1.08) *p=0.79* |
| **CXCR3** | 1.02 (0.89 to 1.17) *p=0.82* | 1.15 (0.94 to 1.41) *p=0.18* | | 1.09 (0.94 to 1.26)  *p=0.25* | **0.97 (0.94 to 1.00) *p=0.046*** | 0.94 (0.88 to 1.00) *p=0.057* | | 0.96 (0.93 to 1.00) *p=0.050* |
| **CXCR5** | 0.95 (0.89 to 1.02) *p=0.18* | **0.88 (0.81 to 0.96) *p=0.0037*** | | **0.93 (0.87 to 0.98)**  ***p=0.013*** | 1.01 (0.95 to 1.07) *p=0.75* | 1.04 (0.93 to 1.17) *p=0.46* | | 1.02 (0.95 to 1.09) *p=0.58* |
| **Adjusted Current CD4** | | | | | | | | |
| **CCR5** | 1.06 (0.95 to 1.18) *p=0.30* | **1.14 (1.01 to 1.29) *p=0.033*** | | ND^4^ | 1.01 (0.96 to 1.05) *p=0.82* | 1.01 (0.93 to 1.09) *p=0.80* | | ND^4^ |
| **CCR6** | 0.98 (0.92 to 1.05) *p=0.61* | **0.88 (0.79 to 0.97) *p=0.0076*** | | ND^4^ | 0.97 (0.90 to 1.05) *p=0.43* | 1.03 (0.87 to 1.21) *p=074* | | ND^4^ |
| **CXCR3** | 1.02 (0.90 to 1.15) *p=0.77* | **1.20 (1.04 to 1.37) *p=0.010*** | | ND^4^ | **0.97 (0.94 to 1.00) *p=0.037*** | **0.92 (0.86 to 0.97) *p=0.0048*** | | ND^4^ |
| **CXCR5** | 0.97 (0.90 to 1.05) *p=0.50* | **0.89 (0.83 to 0.97) *p=0.0068*** | | ND^4^ | 1.01 (0.95 to 1.08) *p=0.67* | 1.07 (0.95 to 1.21) *p=0.26* | | ND^4^ |
| **Adjusted Nadir CD4** | | | | | | | | |
| **CCR5** | 1.08 (0.99 to 1.17) *p=0.076* | **1.15 (1.04 to 1.27) *p=0.0049*** | | ND^4^ | 1.01 (0.96 to 1.05) *p=0.78* | 0.99 (0.90 to 1.08) *p=0.78* | | ND^4^ |
| **CCR6** | 0.97 (0.86 to 1.10) *p=0.64* | **0.80 (0.67 to 0.96) *p=0.015*** | | ND^4^ | 0.97 (0.89 to 1.05) *p=0.39* | 1.04 (0.89 to 1.21) *p=0.66* | | ND^4^ |
| **CXCR3** | 0.99 (0.81 to 1.20) *p=0.91* | 1.25 (0.95 to 1.63) *p=0.11* | | ND^4^ | **0.97 (0.94 to 1.00) *p=0.032*** | **0.94 (0.88 to 1.00) *p=0.049*** | | ND^4^ |
| **CXCR5** | 0.95 (0.89 to 1.02) *p=0.19* | **0.88 (0.81 to 0.96) *p=0.0025*** | | ND^4^ | 1.01 (0.95 to 1.07) *p=0.75* | 1.05 (0.94 to 1.17) *p=0.43* | | ND^4^ |
| **Adjusted Current & Nadir CD4** | | | | | | | | |
| **CCR5** | ND^4^ | ND^4^ | | ND^4^ | 1.01 (0.96 to 1.05) *p=0.82* | 0.99 (0.90 to 1.08) *p=0.80* | | ND^4^ |
| **CCR6** | ND^4^ | ND^4^ | | ND^4^ | 0.96 (0.89 to 1.05) *p=0.37* | 1.05 (0.89 to 1.24) *p=0.59* | | ND^4^ |
| **CXCR3** | ND^4^ | ND^4^ | | ND^4^ | **0.96 (0.93 to 0.99) *p=0.015*** | **0.92 (0.86 to 0.97) *p=0.0056*** | | ND^4^ |
| **CXCR5** | ND^4^ | ND^4^ | | ND^4^ | 1.02 (0.95 to 1.08) *p=0.64* | 1.07 (0.95 to 1.20) *p=0.29* | | ND^4^ |

^1^ Percentage of total memory CD4+ T cells expressing a single chemokine receptor (CKR)

^2^ Lymph node

^3^ Results interpretation: for each 1 unit increase in predictor (percentage of total memory CD4+ T cells expressing single chemokine receptor), the fold change in HIV reservoir outcome [HIV integrated DNA (Int DNA, copies/million CD4 T cells), cell associated-unspliced RNA (CA-US RNA, copies/million CD4+ T cells) or ratio of CA-US RNA:intDNA] in the same tissue is shown. The 95% confidence interval (brackets) and *p* value in *italics* are also shown.

^4^ ND: Not determined.
