# Supplementary material for: Human Immunodeficiency Virus (HIV)–Infected CCR6+ Rectal CD4+ T Cells and HIV Persistence On Antiretroviral Therapy
Source: J Infect Dis. 2019 Dec 4;221(5):744–55. doi: 10.1093/infdis/jiz509 (PMC7026892; doi:10.1093/infdis/jiz509)
Supplement: jiz509_suppl_Supplmentary_Table_5 [file jiz509_suppl_supplmentary_table_5.docx]

**Supplementary Table 5:** Demographics for a subset of 10 people living with HIV on ART that underwent additional large blood draw and rectal pinch biopsy collection.

| **PARAMETER** | **BLOOD and RECTUM**  **n = 10** |
| --- | --- |
| Age, years* | 57.5 (49–61) |
| Gender, n (%): Male  Female  Transgender | 10 (100%) |
|  | 0 (0%) |
|  | 0 (0%) |
| Ethnicity, n (%): Caucasian  African  Hispanic  Asian  Pacific Islander  Mixed | 7 (70%)  2 (20%)  0 (0%)  0 (0%)  1 (10%)  0 (0%) |
| ART, years* | 10.8 (5.6–13.4) |
| Viral Load, copies / ml* | < 40 |
| CD4 T cell count*: Nadir, cells / μl  Current, cells / μl  Current % | 135 (63–337)  578 (456–722)  26 (22–30) |
| CD8 T cell count*: Current, cells / μl | 1020 (888–1175) |
| CD4/CD8 T cell count* | 0.57 (0.48-0.66) |
|  |  |
