# Supplementary material for: Human Immunodeficiency Virus (HIV)–Infected CCR6+ Rectal CD4+ T Cells and HIV Persistence On Antiretroviral Therapy
Source: J Infect Dis. 2019 Dec 4;221(5):744–55. doi: 10.1093/infdis/jiz509 (PMC7026892; doi:10.1093/infdis/jiz509)
Supplement: jiz509_suppl_Supplmentary_Table_6 [file jiz509_suppl_supplmentary_table_6.docx]

**Supplementary Table 6:** Geometric mean fold difference in chemokine mRNA expression between paired rectal and lymph node (LN) tissues from 6 people living with HIV on ART (n=6).

| **Chemokine^1^** | **Geometric mean fold difference^2^** | **Lower CL^2^** | **Upper CL^2^** | **p-value**^3^ |
| --- | --- | --- | --- | --- |
| **CCL5** | **0.337** | **0.210** | **0.541** | **0.002** |
| **CCL20** | **11.369** | **3.279** | **39.423** | **0.004** |
| **CCL19** | **0.033** | **0.013** | **0.082** | **<0.001** |
| **CCL21** | **0.007** | **0.003** | **0.018** | **<0.001** |
| **CXCL9** | **0.144** | **0.082** | **0.253** | **<0.001** |
| **CXCL10** | 0.572 | 0.212 | 1.547 | 0.210 |
| **CXCL11** | 0.353 | 0.099 | 1.259 | 0.089 |
| **CXCL12** | **0.077** | **0.039** | **0.150** | **<0.001** |
| **CXCL13** | **0.095** | **0.030** | **0.303** | **0.003** |

^1^ Relative chemokine mRNA levels were log-transformed and compared using a paired t test. The log-transformed data reduced skewness to better meet the distributional assumptions of the t test.

^2^ The log-transformed values were back-transformed to the fold differences in these columns. The geometric mean fold difference between rectal versus LN tissue is shown. CL: confidence limit.

^3^ All t-tests and their p-values derive from 6 patients with paired specimens.
