# Supplementary material for: Human Immunodeficiency Virus (HIV)–Infected CCR6+ Rectal CD4+ T Cells and HIV Persistence On Antiretroviral Therapy
Source: J Infect Dis. 2019 Dec 4;221(5):744–55. doi: 10.1093/infdis/jiz509 (PMC7026892; doi:10.1093/infdis/jiz509)
Supplement: jiz509_suppl_Supplmentary_Table_7 [file jiz509_suppl_supplmentary_table_7.docx]

**Supplementary Table 7:** Relationship of the HIV reservoir in total CD4+ T cells with chemokine mRNA expression in lymph node and rectal tissues from people living with HIV on ART using negative binomial regression models.

| **Chemokine^1^** | **LN^2^** | | **RECTUM** | | |
| --- | --- | --- | --- | --- | --- |
|  | **Int DNA^3^**  **n=7** | **CA-US RNA^3^**  **n=7** | **Int DNA^3^**  **n=16** | **CA-US RNA^3^**  **n=16** | |
| **Unadjusted** | | | | | |
| **CCL5** | **0.20 (0.05 to 0.84)**  ***p=0.028*** | 0.15 (0.02 to 1.22) *p=0.076* | 1.07 (0.52 to 2.20) *p=0.85* | | 1.81 (0.50 to 6.61) *p=0.37* |
| **CCL20** | 0.30 (0.09 to 1.00) *p=0.050* | 0.26 (0.04 to 1.74) *p=0.17* | 0.97 (0.68 to 1.37) *p=0.85* | | 0.92 (0.41 to 2.05) *p=0.83* |
| **CCL19** | **0.15 (0.05 to 0.50) *p=0.0017*** | **0.05 (0.01 to 0.30) *p=0.0011*** | 1.15 (0.84 to 1.58) *p=0.37* | | 1.25 (0.63 to 2.52) *p=0.52* |
| **CCL21** | 0.33 (0.10 to 1.07) *p=0.064* | **0.10 (0.01 to 0.73) *p=0.024*** | 0.83 (0.61 to 1.12) *p=0.22* | | 0.92 (0.52 to 1.65) *p=0.79* |
| **CXCL9** | 0.88 (0.22 to 3.54) *p=0.86* | 5.10 (0.40 to 64.39) *p=0.21* | 1.19 (0.76 to 1.88) *p=0.44* | | 1.54 (0.82 to 2.90) *p=0.18* |
| **CXCL10** | 0.88 (0.13 to 6.10) *p=0.89* | 1.48 (0.06 to 34.51) *p=0.81* | 1.25 (0.85 to 1.82) *p=0.26* | | 1.95 (0.98 to 3.89) *p=0.057* |
| **CXCL11** | 0.50 (0.12 to 2.07) *p=0.34* | 0.71 (0.05 to 9.73) *p=0.80* | 1.24 (0.90 to 1.72) *p=0.19* | | 1.68 (0.97 to 2.90) *p=0.063* |
| **CXCL12** | 0.64 (0.36 to 1.14) *p=0.13* | 0.45 (0.17 to 1.16) *p=0.10* | 0.86 (0.34 to 2.14) *p=0.74* | | 0.28 (0.03 to 2.49) *p=0.25* |
| **CXCL13** | 1.29 (0.57 to 2.95) *p=0.54* | **3.94 (1.11 to 14.04) *p=0.034*** | 1.05 (0.81 to 1.35) *p=0.73* | | 1.21 (0.79 to 1.85) *p=0.39* |
| **Adjusted Current CD4** | | | | | |
| **CCL5** | **0.21 (0.05 to 0.89)**  ***p=0.034*** | 0.15 (0.02 to 1.10) *p=0.062* | 1.08 (0.52 to 2.21) *p=0.85* | | 2.02 (0.56 to 7.23) *p=0.28* |
| **CCL20** | **0.28 (0.09 to 0.89) *p=0.031*** | 0.19 (0.03 to 1.18) *p=0.074* | 0.89 (0.58 to 1.39) *p=0.62* | | 0.97 (0.40 to 2.36) *p=0.94* |
| **CCL19** | **0.16 (0.03 to 0.90) *p=0.037*** | **0.06 (0.003 to 0.93) *p=0.044*** | 1.15 (0.83 to 1.59) *p=0.40* | | 1.24 (0.58 to 2.63) *p=0.58* |
| **CCL21** | 0.48 (0.12 to 1.87) *p=0.29* | 0.20 (0.01 to 2.85) *p=0.23* | 0.80 (0.59 to 1.09) *p=0.16* | | 0.92 (0.51 to 1.66) *p=0.78* |
| **CXCL9** | 0.63 (0.24 to 1.62) *p=0.34* | 1.45 (0.11 to 19.43) *p=0.78* | 1.19 (0.76 to 1.86) *p=0.45* | | 1.54 (0.81 to 2.93) *p=0.19* |
| **CXCL10** | 0.34 (0.07 to 1.70) *p=0.19* | 0.10 (0.01 to 1.04) *p=0.054* | 1.24 (0.85 to 1.82) *p=0.27* | | 1.95 (0.98 to 3.89) *p=0.059* |
| **CXCL11** | 0.39 (0.11 to 1.45) *p=0.16* | 0.53 (0.03 to 9.07) *p=0.66* | 1.24 (0.89 to 1.72) *p=0.20* | | 1.69 (0.96 to 2.97) *p=0.067* |
| **CXCL12** | 0.78 (0.41 to 1.49) *p=0.45* | 0.64 (0.19 to 2.11) *p=0.46* | 0.86 (0.35 to 2.13) *p=0.75* | | 0.28 (0.03 to 2.61) *p=0.26* |
| **CXCL13** | 1.10 (0.52 to 2.32) *p=0.80* | 3.04 (0.93 to 9.94) *p=0.066* | 1.03 (0.78 to 1.38) *p=0.82* | | 1.22 (0.79 to 1.87) *p=0.37* |
| **Adjusted Nadir CD4** | | | | | |
| **CCL5** | **0.18 (0.04 to 0.83)**  ***p=0.028*** | 0.09 (0.01 to 1.05) *p=0.055* | 1.07 (0.52 to 2.20) *p=0.85* | | 1.92 (0.52 to 7.14) *p=0.33* |
| **CCL20** | 0.27 (0.07 to 1.02) *p=0.054* | 0.14 (0.01 to 1.75) *p=0.13* | 0.97 (0.68 to 1.37) *p=0.85* | | 1.05 (0.43 to 2.55) *p=0.92* |
| **CCL19** | **0.08 (0.03 to 0.21) *p<0.0001*** | **0.02 (0.004 to 0.13) *p<0.0001*** | 1.12 (0.85 to 1.66) *p=0.31* | | 1.26 (0.63 to 2.54) *p=0.51* |
| **CCL21** | **0.23 (0.07 to 0.76) *p=0.02*** | **0.07 (0.01 to 0.51) *p=0.0089*** | 0.83 (0.61 to 1.12) *p=0.22* | | 0.96 (0.53 to 1.73) *p=0.89* |
| **CXCL9** | 0.89 (0.23 to 3.38) *p=0.86* | 5.70 (0.63 to 51.89) *p=0.12* | 1.20 (0.76 to 1.90) *p=0.43* | | 1.56 (0.82 to 2.99) *p=0.18* |
| **CXCL10** | 1.03 (0.14 to 7.30) *p=0.98* | 6.89 (0.19 to 256.2) *p=0.30* | 1.30 (0.87 to 1.92) *p=0.20* | | 1.93 (0.94 to 3.99) *p=0.074* |
| **CXCL11** | 0.52 (0.11 to 2.54) *p=0.42* | 1.50 (0.03 to 88.78) *p=0.84* | 1.29 (0.92 to 1.81) *p=0.13* | | 1.70 (0.97 to 2.98) *p=0.063* |
| **CXCL12** | 0.63 (0.35 to 1.13) *p=0.12* | 0.45 (0.17 to 1.18) *p=0.10* | 0.86 (0.34 to 2.15) *p=0.74* | | 0.27 (0.03 to 2.36) *p=0.24* |
| **CXCL13** | 1.25 (0.45 to 3.48) *p=0.67* | **4.67 (1.13 to 19.28) *p=0.033*** | 1.06 (0.80 to 1.41) *p=0.67* | | 1.22 (0.80 to 1.86) *p=0.35* |
| **Adjusted Current & Nadir CD4** | | | | | |
| **CCL5** | ND^4^ | ND^4^ | 1.07 (0.52 to 2.22) *p=0.85* | | 2.14 (0.61 to 7.49) *p=0.23* |
| **CCL20** | ND^4^ | ND^4^ | 0.88 (0.57 to 1.38) *p=0.59* | | 1.33 (0.48 to 3.67) *p=0.59* |
| **CCL19** | ND^4^ | ND^4^ | 1.18 (0.84 to 1.65) *p=0.33* | | 1.21 (0.57 to 2.59) *p=0.62* |
| **CCL21** | ND^4^ | ND^4^ | 0.80 (0.59 to 1.09) *p=0.15* | | 0.96 (0.52 to 1.75) *p=0.89* |
| **CXCL9** | ND^4^ | ND^4^ | 1.20 (0.77 to 1.88) *p=0.43* | | 1.54 (0.79 to 3.01) *p=0.21* |
| **CXCL10** | ND^4^ | ND^4^ | 1.30 (0.87 to 1.92) *p=0.20* | | 1.91 (0.92 to 3.98) *p=0.084* |
| **CXCL11** | ND^4^ | ND^4^ | 1.31 (0.93 to 1.83) *p=0.12* | | 1.69 (0.95 to 3.01) *p=0.074* |
| **CXCL12** | ND^4^ | ND^4^ | 0.87 (0.35 to 2.16) *p=0.76* | | 0.28 (0.03 to 2.57) *p=0.26* |
| **CXCL13** | ND^4^ | ND^4^ | 1.05 (0.77 to 1.43) *p=0.75* | | 1.24 (0.82 to 1.90) *p=0.31* |

^1^ Relative Chemokine mRNA

^2^ Lymph node

^3^ Results interpretation: for each 2-fold increase in predictor (relative chemokine mRNA), the fold change in HIV reservoir outcome [HIV integrated DNA (Int DNA) or cell associated-unspliced RNA (CA-US RNA) copies/million CD4 T cells] for the same tissue is shown. The 95% confidence interval (brackets) and *p* value in *italics* are also shown.

^4^ ND: Not determined due to low sample number.
