# Supplementary material for: Human Immunodeficiency Virus (HIV)–Infected CCR6+ Rectal CD4+ T Cells and HIV Persistence On Antiretroviral Therapy
Source: J Infect Dis. 2019 Dec 4;221(5):744–55. doi: 10.1093/infdis/jiz509 (PMC7026892; doi:10.1093/infdis/jiz509)
Supplement: jiz509_suppl_Supplmentary_text [file jiz509_suppl_supplmentary_text.doc]

**Supplementary Text:**

HIV-infected CCR6+ Rectal CD4+ T-cells contribute significantly to HIV persistence on antiretroviral therapy

**Supplementary Methods:**

**Includes:**

1. Sample collection and processing
2. HIV reservoir analysis
3. Integrated HIV DNA analysis
4. Cell-associated unspliced RNA analysis
5. Single genome amplification, sequencing and analyses of HIV *env* genes
6. Calculating the relative contribution of CKR expressing subsets to the total HIV integrated DNA reservoir in blood or rectal tissue

C. CKR immunophenotyping

D. Chemokine and T-cell Transcription Factor mRNA analysis

1. Chemokine mRNA analysis in whole tissue
2. Transcription factor mRNA analysis in sorted T-cells

E. Statistical Analysis

**A. Sample collection and processing**

Individuals initially donated blood (n=48), 20 rectal pinch biopsies (n=20) by sigmoidoscopy and/or had an inguinal LN surgically removed (n=8). A portion of whole blood was used for CKR immunophenotyping. From the remaining blood, peripheral blood mononuclear cells were isolated using Ficoll Histopaque-1077 (Sigma-Aldrich, USA) and total CD4+ T-cells then isolated by magnetic negative selection (Stem Cell Technologies, USA). Two rectal biopsies or a 10% LN slice were stored in Allprotect Tissue Reagent (Qiagen, Germany) at -80oC for chemokine analysis. The remaining tissue was processed for rectal mononuclear cells using Liberase digestion or LN mononuclear cells via mechanical disruption, and tissue mononuclear cells used for CKR immunophenotyping (see section B below) and total CD4+ T-cell isolation as published [1-5].

Ten individuals had a second sigmoidoscopy and collection of 150ml blood for analysing HIV in sorted CCR6/CXCR3 T-cell subsets. Thirty rectal pinch biopsies collected via sigmoidoscopy were stored in RPMI-1640 supplemented with 10% foetal bovine serum for transport on ice and immediately processed into rectal mononuclear cells using epithelial stripping and liberase enzyme digestion (to preserve CKR staining) [5]. Large 150ml blood draws collected into EDTA tubes were pooled, depleted of CD8+ T-cells using RosetteSep Human CD8 Depletion Cocktail as per manufacturer instructions with half the recommended volume of CD8 Depletion Rosette Sep cocktail (Stem Cell Technologies, USA), and the CD8-depleted PBMCs collected via Ficoll-Histopaque-1077 (Sigma-Aldrich, Missouri USA), washed in PBS and counted.

Freshly isolated rectal single mononuclear cells or CD8-depleted PBMCs were stained with LIVE/DEAD Fixable Aqua Dead Cell Stain (Invitrogen) and the following panel of anti-human antibodies: CD3-Brilliant Violet 711 (OKT-3, BioLegend), CD8-Brilliant Violet 605 (RPA-T8, BioLegend), CD4-APC-eFluor 780 (OKT-4, eBioscience/Thermo Fisher Scientific), CD45RA-PE-Cy7 (HI100, Becton Dickinson [BD]), CD27-Brilliant Violet 421 (O323, BioLegend), CCR6 (CD196)-PE (11A9, BD), CXCR3 (CD183)-PE-Cy5 (1C6, BD), CD14-V500 (M5E2, BD) and CD45-FITC (2D1, BD) [BioLegend USA; Thermo Fisher Scientific USA; Becton Dickinson and Company (BD) USA; Invitrogen USA]. Cells were stained at 37oC for 15 min and washed once in FACS buffer (PBS with 0.5% bovine serum albumin and 1mM ethylenediaminetetraacetic acid (EDTA). Fluorescence minus one (FMO) controls were prepared to set gates. Approximately 1 million events were collected for phenotype analysis and the sample was sorted on an ARIA FACS sorter [Becton Dickinson (BD), USA] for the single cell, Aqua- live cell, CD14-, CD45+, CD3+, CD8lo, CD45RA- population into either: the CCR6+CXCR3+, CCR6+CXCR3-, CCR6-CXCR3+ and CCR6-CXCR3- subsets for blood; or the CCR6+CXCR3+ and pooled non-CCR6+CXCR3+ subset (CCR6+CXCR3-, CCR6-CXCR3+ and CCR6-CXCR3- cells due to low numbers of these cells) for rectal tissue.

Sorted cells were stored at -80oC as a frozen cell pellet or in 1ml RNAprotect Cell Reagent (Qiagen, Germany) for DNA or RNA analysis respectively.

**B. HIV reservoir analysis**

1. **Integrated HIV DNA analysis**

Integrated HIV DNA was measured using Alu-LTR quantitative PCR (qPCR), where the first-round PCR contains primers to Alu repeat elements in the host genome and the viral LTR, and the second-round PCR includes primers to the LTR only as published [6, 7]. This assay had a lower detection limit of 1 copy per PCR reaction. Zero copies was reported for no qPCR signal while detectable signal <1 copy was assigned 0.5 copies.

1. **Cell-associated unspliced RNA analysis**

Cell-associated unspliced RNA (CA-US RNA) was measured via reverse transcription qPCR (RT-qPCR) as published [8]. This assay had a lower detection limit of 1 copy per PCR reaction. Zero copies was reported for no qPCR signal while detectable signal <1 copy was assigned 0.5 copies.

1. **Single genome amplification, sequencing and analyses of HIV *env***

**genes**

Genomic DNA (gDNA) was isolated from CD4+ T-cells from peripheral blood, LN or rectal tissue using an AllPrep DNA/RNA Micro or Mini kit (Qiagen, Germany) according to manufacturer instructions. The gDNA was then used for single genome amplification of the HIV *env* gp160 gene as published using a nested PCR with Platinum Taq High Fidelity polymerase (Invitrogen) with the following modifications [9]. The first round PCR used the primers: envB5out (5’-TAGAGCCCTGGAAGCATCCAGGAAG-3’) and envB3out (5’- TTGCTACTTGTGATTGCTCCATGT-3’). Then, 1ul of first round PCR product was transferred to the second round PCR and amplified using the primers: envB5in (5’-CACCTTAGGCATCTCCTATGGCAGGAAGAAG-3’, the underlined four nucleotides allow for TOPO directional cloning) and envB3in (5’-GTCTCGAGATACTGCTCCCACCC-3’). For single copy amplification, the nested PCR was first performed using serially diluted gDNA to identify a dilution in which 2 of 8 nested PCRs were positive indicative of single genome amplification. This gDNA dilution was selected and the nested PCR performed again for an entire 96 well plate. 1.2% agarose gel electrophoresis was used to identify positive PCRs. Positive 3kb PCR products were then purified (QIAquick PCR Purification Kit, Qiagen, Germany) and directly Sanger sequenced (Australian Genome Research Facility, Australia) using 3 different primers: forward primer ED5 (5’-ATGGGATCAAAGCCTAAAGCCATGTG-3’) or reverse primers ED12 (5’- AGTGCTTCCTGCTGCTCCCAAGAACCCAAG-3’) or V3R3 (5’-TACAATTTCTGGGTCCCCTCCTG-3’).

The *env* sequences were first examined for hypermutations using Hypermut2.0 (hiv.lanl.gov) and hypermutated sequences removed from further analysis. Phylogenetic analyses of HIV *env* sequences from individual patients were performed using maximum likelihood trees generated using MEGA 6 ([http://megasoftware.net](http://megasoftware.net/)). The general time reversible plus gamma model of nucleotide substitution was used as selected by the Los Alamos National Laboratory FindModel program (hiv.lanl.gov). 100 bootstrap replicates were used to statistically support the phylogenetic tree structures. Average pairwise distances were calculated in MEGA 6 to identify identical sequences, which were confirmed by individual sequence alignments. This sequence approach had a clonal prediction score of 83%, which gives 83% confidence that an identical *env* sequence is an identical provirus [10].

Genetic compartmentalization was assessed in anatomical sites using either a Simmonds Association Index (10 relabellings per sample, 1000 bootstraps, proportion of reshuffling=2) or Wright’s Measure of Population Subdivison (maximum likelihood method, General Time reversible method, 1000 relabellings, K_ST_ estimate) [11-13]. The Simmonds Association Index measures the degree of population structure in the phylogenetic tree by weighting the contribution of each internal node based on its depth in the tree, and determining the significance of the observed value using a bootstrap sample over the structure of the population as well as the shape of the phylogenetic tree. In contrast, the Wright’s Measure of Population Subdivison compares the mean pairwise genetic distance between 2 sequences sampled from different compartments to the mean distance between sequences sampled from the same compartment, with statistical significance determined using a population-structure randomization test [11].

1. **Calculating the contribution of CKR expressing subsets to the total HIV integrated DNA reservoir in blood or rectal tissue**

The relative contribution of a CKR expressing T-cell subset to the total pool of memory T-cells harbouring integrated HIV DNA in blood or rectal tissue was estimated as published [14] using the equation: [relative pool of HIV-infected cells per subset ie. (HIV integrated DNA copies per 10^6^ cells for each subset x subset frequency) / 100 ] / [sum of the relative pool of HIV-infected cells in all subsets] x 100. Of note, for rectal tissue calculations in Figure 4E, the relative contribution of the CCR6+CXCR3-, CCR6-CXCR3+ and CCR6-CXCR3- rectal T-cell subsets was calculated using their frequencies together with integrated HIV DNA in the non CCR6+CXCR3+ T-cell pool to determine their relative contribution to the total pool of infected cells.

**C. CKR immunophenotyping**

Whole peripheral blood samples were directly stained for CKR expression on total memory CD4+ T-cells as published [3]. Single mononuclear cells freshly isolated from LN or rectal biopsies were stained with a CKR panel containing the anti-human antibodies: CD3-Alexa700 (BD #557943), CD4-Qdot605 (Invitrogen #Q10008), CD45RA-APC-H7 (BD #560674), CD27-BV650 (BioLegend #302827), CCR7-PE-Cy7 (BD #557648), CD14-V500 (BD #561391), CD19-AmCyan (BD #339190), CCR5 (CD195)-V450 (BD Horizon #562121), CCR6 (CD196)-PE (BD #559562), CXCR3 (CD183)-PE-Cy5 (BD #551128) and CXCR5-Alexa488 (BD #558112) [BD USA; Invitrogen USA; BioLegend USA]. Samples were analysed using an LSRII Flow Cytometer (BD, USA) and FlowJo software (FlowJo, LLC Oregon USA). CCR7 was excluded from analysis due to lost staining intensity over processing donors. CKR expression was assessed on total memory CD4+ T-cells that included CD45RA-CD27+ central/transitional, CD45RA-CD27- effector and CD45RA+CD27- terminally differentiated subsets. However, CD45RA+CD27+ naïve cells were excluded from analysis as rectal tissue lacks naïve cells, which might impact comparison to blood and LN sites that harbour naïve cells.

**D. Chemokine and T-cell Transcription Factor mRNA analysis**

**i) Chemokine mRNA analysis in whole tissue**

RNA was extracted from 2 rectal pinch biopsies or the LN slice stored in Allprotect Tissue Reagent (Qiagen, Germany) using the RNeasy Lipid Mini RNA extraction kit (Qiagen, Germany) according to manufacturer instructions. This included: homogenising each tissue sample in 1ml QIAzol using a TissueRuptor (Qiagen, Germany) with a new disposable probe, and two optional on-column RNase-free DNase treatments (Qiagen, Germany). RNA was typically eluted in 150ul RNase-free H_2_O and 5ul single use aliquots stored at -80oC. RNA concentration and quality were measured using a NanoDrop 1000 as per manufacturer instructions (Thermo Fisher Scientific, Massachusetts USA).

The expression of chemokine mRNA was analysed in tissue RNA samples via RT-qPCR using the relative standard curve quantitation method that compares mRNA expression in test samples to a calibrator sample (Applied Biosystems: Guide to Performing Relative Quantitation of Gene Expression Using Real-time Quantitative PCR). For reverse transcription reactions, 100 ng of RNA tissue sample, 10 fold dilutions of standard (100, 10, 1, 0.1, 0.01 or 0.001 ng RNA), and untreated PBMC RNA calibrator (most RT-qPCRs) or human liver RNA calibrator (CCL21 RT-qPCR as CCL21 was not expressed in PBMC) were used. For each RT-qPCR type, different standards were selected depending on the abundance of the target RNA as follows: 18S rRNA RT-qPCR - untreated PBMC RNA; CXCL9, CXCL10 and CXCL11 RT-qPCR - RNA from PBMC treated 24 hours with 0.1 ug/ml IFN-gamma and 2.5 ng/ml TNF-alpha; CCL20 RT-qPCR – RNA from PBMC treated 2 hours with 100 ng/ml lipopolysaccharide (LPS); CCL5, CCL19 and CXCL13 RT-qPCR - RNA from PBMC treated 24 hours with 100 ng/ml LPS; and CXCL12 and CCL21 RT-qPCR - human liver tissue RNA.

For reverse transcription reactions, RNA was reverse transcribed into cDNA in 20 μl reactions using Superscript III reverse transcriptase (RT, Thermo Fisher Scientific, USA) with modifications to the manufacturer protocol. No RT controls were run for each sample to ensure that there was no residual DNA contamination in RNA samples. For each reaction, the appropriate RNA amount was made up to 11.5 μl with water and 1 μl of 10mM dNTP, 0.5 μl of 50 μM random hexamers and 0.5 μl of 0.5 μg/μl Oligo dT added. Primers were annealed for 65^o^C, 5 min followed by ice ≥ 2 min. Then, 4 μl of 5x First Strand buffer, 1 μl of 0.1M DTT, 0.5 μl of 40U/μl RNaseOUT recombinant ribonuclease inhibitor and 1 μl of Superscript III (or 1 μl of water for no RT controls) were added. Reactions were reverse transcribed into cDNA at 42^o^C/45 min, the enzymes heat inactivated at 80^o^C/15 min and cDNA stored at -80^o^C when not in use.

2 μl of neat or diluted cDNA was then analysed for various chemokine mRNA or an 18S rRNA housekeeping gene control using Applied Biosystems (Thermo Fisher Scientific, USA) reagents in a 20 μl total reaction with 1x Universal Master Mix II (no UNG) and 1x FAM TaqMan probe. The following human RNAs (with TaqMan probe assay codes) were assessed: CCL5 (Hs00174575_m1), CCL19 (Hs00171149_m1), CCL20 (Hs01011368_m1), CCL21 (Hs99999110_m1), CXCL9 (Hs00171065_m1), CXCL10 (Hs00171042_m1), CXCL11 (Hs00171138_m1), CXCL12 (Hs00171022_m1), CXCL13 (Hs00757930_m1) and 18S rRNA (Hs99999901_s1) to normalise for cell input. To fit in the standard curve, 2 ul of **neat** cDNA was used for reactions except for: **1:10** diluted cDNA for all 18S rRNA samples, CCL19 rectal, CXCL12 rectal and CXCL13 rectal samples; or **1:100** diluted cDNA for CCL19 LN, CCL20 rectal, CCL21 rectal, CXCL12 LN and CXCL13 LN samples; or **1:1000** for CCL21 LN samples. Reactions were assessed using a Stratagene Mx3000P qPCR system (Agilent Technologies, Germany) and associated MxPro software using the qPCR cycling conditions: 1 cycle of 95^o^C/10 min and then multiple cycles of 95^o^C/15 sec and 60^o^C/1 min depending on the RNA product being amplified ie. 40 cycles – 18S rRNA; 50 cycles – CCL5, CCL19, CXCL9, CXCL10, CXCL11, CXCL13; 60 cycles – CCL20, CCL21, CXCL12. All reactions with reverse transcriptase (RT+) were assessed in triplicate qPCR while no RT (RT-) controls had one qPCR replicate. Amounts of target RNA (ng) in the samples were analysed using the default standard curve generated by the MxPro qPCR software, ng amounts were multiplied by a dilution factor if applicable and then ng amounts were averaged across triplicate RT+ samples. The following equation was then used to assess the relative expression of chemokine RNA in the tissue sample versus the calibrator control: (tissue: ng chemokine RNA / ng 18S rRNA) / (calibrator: ng chemokine RNA / ng 18S rRNA).

To rule out any impact of RT-qPCR inhibitors in tissue RNA samples from the extraction method, tissue RNA samples were also analysed using a Solaris RNA Spike Control Kit (Millennium Science, Thermo Scientific, USA). 100ng RNA tissue samples, 100 ng untreated PBMC calibrator, or water only as the no inhibitor control, were reverse transcribed as above with the following exception. When reverse transcription reactions were assembled, the RNA was resuspended in 9.5 ul of water (or water only for the water control) and 2 μl of 10x Solaris artificial Spike RNA then added for a 1x final concentration of Solaris Spike RNA in the 20ul RT reaction. Samples without RT were included as controls. 2.5 μl cDNA was assessed for Solaris Spike RNA in a 25 μl qPCR reaction containing 1.25 μl Solaris Spike TaqMan FAM probe in the kit and 1x Universal Master Mix II (no UNG) (Applied Biosystems, Thermo Fisher Scientific, USA). Reactions were assessed using a Stratagene Mx3000P qPCR system (Agilent Technologies, Germany) and associated MxPro software with the qPCR cycling conditions: 1 cycle of 95^o^C/10 min and 40 cycles of 95^o^C/15 sec and 60^o^C/1 min. Samples with reverse transcriptase (RT+) were assessed in triplicate qPCR while no RT (RT-) controls had one qPCR replicate. For RT+ samples, the Ct of the tissue or calibrator sample was subtracted from the Ct value of the water control to assess potential RT-qPCR inhibition. Ct changes <2 were considered no significant inhibition. The tissue and calibrator samples had no significant inhibition (data not shown).

**ii) Transcription factor mRNA analysis in sorted T-cells**

To analyse transcription factor mRNA in blood CCR6 and/or CXCR3 sorted CD4+ T-cell subsets or rectal total CD4+ T-cells, RNA was extracted from the sorted T-cells using a Qiagen AllPrep DNA/RNA Micro (≤ 500,000 cells) or Mini (> 500,000 cells) Kit according to manufacturer instructions. This included: homogenising samples through a QIAshredder and for the RNA extraction, performing two on-column RNase-free DNase digests as per manufacturer instructions, and eluting the RNA in 50ul RNase-free H_2_O (Qiagen Germany). For the calibrator sample, RNA was extracted from untreated PBMC isolated from an uninfected healthy donor using the same method. Single use RNA aliquots were stored at -80oC for all samples for subsequent RT-qPCRs.

The expression of RORγT, FoxP3 and T-bet mRNA in extracted T-cell RNAs were analysed by RT-qPCR using the same procedure above for chemokine mRNA analysis except for the following changes. 11.5 μl of RNA from T-cell samples or 1x10^5^ untreated PBMC calibrator were added to reverse transcription reactions. Dilutions of RNA (100, 25, 6.25, 1.56, 0.78 and 0.098 ng) from healthy donor PBMCs stimulated 3 days with 10 μg/ml PHA and 10 U/ml IL-2 were used to generate the standard curve. 2 μl of undiluted cDNA or 1:10 diluted cDNA where necessary to fit on the standard curve, was added to 20 μl qPCR reactions and assessed for the following human RNAs using FAM TaqMan probes (Applied Biosystems, Thermo Fisher Scientific USA): RORγT/RORC (Hs01076112_m1), FoxP3 (Hs01085834_m1), T-bet/TBX21 (Hs00203436_m1) and 18S rRNA (Hs99999901_s1) to normalise for cell input. qPCR reactions were analysed on the Stratagene Mx3000P qPCR system (Agilent Technologies, Germany) using associated MxPro software and cycling conditions: 1 cycle of 95^o^C/10 min and then 60 cycles of 95^o^C/15 sec and 60^o^C/1 min. RT+ reactions were assessed in duplicate qPCR while RT- controls had one qPCR replicate. Amounts of target RNA (ng) in samples were analysed using the default standard curve generated by the MxPro qPCR software, ng amounts were multiplied by a dilution factor if applicable and then ng amounts of target were averaged across duplicate RT+ samples. The following equation was then used to assess the relative expression of transcription factor RNA in the T-cell samples versus the PBMC calibrator control: (T-cell: ng transcription factor RNA / ng 18S rRNA) / (untreated PBMC calibrator: ng transcription factor RNA / ng 18S rRNA).

**E. Statistical Analysis**

Data distributions were analysed using descriptive statistics and scatter plots (Prism version 6). Spearman correlation assessed relationships between HIV reservoir measures (HIV integrated DNA, CA-USRNA, or ratio of CA-USRNA:integrated DNA) within or between peripheral blood, LN or rectal total CD4+ T-cells (Prism version 6). The Wilcoxon matched-pairs signed rank test assessed differences in: percentages of CKR-expressing cell types in tissue sites; HIV integrated DNA in CCR6 and/or CXCR3 T-cell subsets and the contribution of these subsets to the total HIV integrated DNA reservoir in blood or rectum; and transcription factor expression in blood T-cell subsets (*P* values <0.05 were marked with asterisk/s, Prism version 6).

For 6 participants with paired rectal and LN samples, relative chemokine mRNA levels were log transformed (to reduce skewness to better meet the distributional assumption of the t test) and then compared using a paired t test. Comparisons having a *P* value < 0.05 were marked with asterisk/s or in bold text. The geometric mean fold-difference in chemokine levels between paired tissues was also determined with upper and lower confidence levels shown (Stata version 13.1, Stata Corp, Texas, USA).

Negative binomial regression models were used to assess relationships between frequencies of CKR-expressing cells or relative chemokine mRNA levels, as predictors, with measures of the HIV reservoir (HIV integrated DNA, CA-USRNA or ratio of CA-USRNA:integrated DNA) as the outcome within the same tissue site. This method was selected for multiple reasons as previously published [3, 15, 16]. First, it allowed for additional adjustment for the potentially confounding effects of current CD4+ T-cell count (cells/μl), nadir CD4+ T-cell count (cells/μl) or both, which are known to influence HIV reservoir levels [3, 17-19]. Second, it allowed for the lower precision of HIV copies per input nucleic acid in some samples (due to the number of HIV copies and/or input into the assay being low) to be accounted for. Third, it allowed the accurate use of zero copies of HIV reservoir in samples without resorting to ad hoc modifications to allow taking logarithms. Finally, this method was selected to match other analyses from the same participants [3, 4, 15].

Negative binomial regression was also used to assess relationships between HIV reservoir values in blood, LN and rectal tissues. For comparing HIV integrated DNA or CA-US RNA between paired tissues, tissue type was used as the predictor, reservoir copies as the outcome, and reservoir assay input as an offset. For comparing the ratio of HIV CA-US RNA:integrated DNA between paired tissues, mixed effects negative binomial regression models were used, with reservoir type (CA-US RNA versus DNA), tissue type and an interaction term between reservoir and tissue type as predictors, reservoir copies as the outcome, and reservoir assay input as an offset.

While many parameters were compared using negative binomial regression, nominal p-values were reported without adjustment for multiple comparisons for reasons previously outlined using other data from the same participants [15]. This approach was used because standard methods of such adjustment would be focused on avoiding one or more results with p<0.05 in the case where all differences are truly zero [20-22]. However, this is an unrealistic hypothesis about the state of nature in our study. Adjustment would also require that each result detract from the others. However, there are clear biological relationships between many associations that are examined, and these permit coherent sets of findings to reinforce each other rather than detract from one another. Therefore, we consider multiple comparison adjustment not appropriate for results in this paper and thus multiple comparison adjustment was not performed [23]. These analyses were run using Stata version 13.1 (Stata Corp, Texas, USA).

**Supplementary References:**

1. Josefsson L, von Stockenstrom S, Faria NR, et al. The HIV-1 reservoir in eight patients on long-term suppressive antiretroviral therapy is stable with few genetic changes over time. Proceedings of the National Academy of Sciences of the United States of America **2013**; 110:E4987-96.

2. von Stockenstrom S, Odevall L, Lee E, et al. Longitudinal Genetic Characterization Reveals That Cell Proliferation Maintains a Persistent HIV Type 1 DNA Pool During Effective HIV Therapy. The Journal of infectious diseases **2015**; 212:596-607.

3. Khoury G, Anderson JL, Fromentin R, et al. Persistence of integrated HIV DNA in CXCR3 + CCR6 + memory CD4+ T cells in HIV-infected individuals on antiretroviral therapy. AIDS **2016**; 30:1511-20.

4. Khoury G, Fromentin R, Solomon A, et al. Human Immunodeficiency Virus Persistence and T-Cell Activation in Blood, Rectal, and Lymph Node Tissue in Human Immunodeficiency Virus-Infected Individuals Receiving Suppressive Antiretroviral Therapy. The Journal of infectious diseases **2017**; 215:911-9.

5. Trapecar M, Khan S, Roan NR, et al. An Optimized and Validated Method for Isolation and Characterization of Lymphocytes from HIV+ Human Gut Biopsies. AIDS research and human retroviruses **2017**; 33:S31-s9.

6. Vandergeeten C, Fromentin R, Merlini E, et al. Cross-clade ultrasensitive PCR-based assays to measure HIV persistence in large-cohort studies. Journal of virology **2014**; 88:12385-96.

7. Sonza S, Maerz A, Deacon N, Meanger J, Mills J, Crowe S. Human immunodeficiency virus type 1 replication is blocked prior to reverse transcription and integration in freshly isolated peripheral blood monocytes. Journal of virology **1996**; 70:3863-9.

8. Elliott JH, Wightman F, Solomon A, et al. Activation of HIV transcription with short-course vorinostat in HIV-infected patients on suppressive antiretroviral therapy. PLoS pathogens **2014**; 10:e1004473.

9. Salazar-Gonzalez JF, Bailes E, Pham KT, et al. Deciphering human immunodeficiency virus type 1 transmission and early envelope diversification by single-genome amplification and sequencing. Journal of virology **2008**; 82:3952-70.

10. Laskey SB, Pohlmeyer CW, Bruner KM, Siliciano RF. Evaluating Clonal Expansion of HIV-Infected Cells: Optimization of PCR Strategies to Predict Clonality. PLoS pathogens **2016**; 12:e1005689.

11. Zarate S, Pond SL, Shapshak P, Frost SD. Comparative study of methods for detecting sequence compartmentalization in human immunodeficiency virus type 1. Journal of virology **2007**; 81:6643-51.

12. Wang TH, Donaldson YK, Brettle RP, Bell JE, Simmonds P. Identification of shared populations of human immunodeficiency virus type 1 infecting microglia and tissue macrophages outside the central nervous system. Journal of virology **2001**; 75:11686-99.

13. Hudson RR, Boos DD, Kaplan NL. A statistical test for detecting geographic subdivision. Mol Biol Evol **1992**; 9:138-51.

14. Gosselin A, Wiche Salinas TR, Planas D, et al. HIV persists in CCR6+CD4+ T cells from colon and blood during antiretroviral therapy. AIDS **2017**; 31:35-48.

15. Fromentin R, Bakeman W, Lawani MB, et al. CD4+ T Cells Expressing PD-1, TIGIT and LAG-3 Contribute to HIV Persistence during ART. PLoS pathogens **2016**; 12:e1005761.

16. Elliott JH, McMahon JH, Chang CC, et al. Short-term administration of disulfiram for reversal of latent HIV infection: a phase 2 dose-escalation study. The lancet HIV **2015**; 2:e520-9.

17. Boulassel MR, Chomont N, Pai NP, Gilmore N, Sekaly RP, Routy JP. CD4 T cell nadir independently predicts the magnitude of the HIV reservoir after prolonged suppressive antiretroviral therapy. Journal of clinical virology **2012**; 53:29-32.

18. Chun TW, Justement JS, Pandya P, et al. Relationship between the size of the human immunodeficiency virus type 1 (HIV-1) reservoir in peripheral blood CD4+ T cells and CD4+:CD8+ T cell ratios in aviremic HIV-1-infected individuals receiving long-term highly active antiretroviral therapy. The Journal of infectious diseases **2002**; 185:1672-6.

19. Chomont N, El-Far M, Ancuta P, et al. HIV reservoir size and persistence are driven by T cell survival and homeostatic proliferation. Nature medicine **2009**; 15:893-900.

20. Rothman K. No adjustments are needed for multiple comparisons. Epidemiology **1990**; 1:43-6.

21. Savitz D, Olshan A. Multiple comparisons and related issues in the interpretation of epidemiologic data. Am J Epidemiol **1995**; 142:904-8.

22. Perneger T. What's wrong with Bonferroni adjustments. BMJ **1998**; 316:1236-8.

23. Bacchetti P. Peer review of statistics in medical research: the other problem. BMJ **2002**; 324:1271-3.
